# A statistical approach to estimating the strength of cell-cell interactions under the differential adhesion hypothesis

**DOI:** 10.1186/1742-4682-4-37

**Published:** 2007-09-18

**Authors:** Mathieu Emily, Olivier François

**Affiliations:** 1 TIMC-TIMB, Université Joseph Fourier, INP Grenoble, Faculty of Medicine, 38706 La Tronche cedex, France; 2 Bioinformatics Research Center (BiRC), University of Aarhus, Hoegh-Guldbergs Gade 10, 8000 Aarhus C, Denmark

## Abstract

**Background:**

The Differential Adhesion Hypothesis (DAH) is a theory of the organization of cells within a tissue which has been validated by several biological experiments and tested against several alternative computational models.

**Results:**

In this study, a statistical approach was developed for the estimation of the strength of adhesion, incorporating earlier discrete lattice models into a continuous marked point process framework. This framework allows to describe an ergodic Markov Chain Monte Carlo algorithm that can simulate the model and reproduce empirical biological patterns. The estimation procedure, based on a pseudo-likelihood approximation, is validated with simulations, and a brief application to medulloblastoma stained by beta-catenin markers is given.

**Conclusion:**

Our model includes the strength of cell-cell adhesion as a statistical parameter. The estimation procedure for this parameter is consistent with experimental data and would be useful for high-throughput cancer studies.

## Background

The development and the maintenance of multi-cellular organisms are driven by permanent rearrangements of cell shapes and positions. Such rearrangements are a key step for the reconstruction of functional organs [1]. *In vitro *experiments such as Holtfreter's experiments on the pronephros [2] and the famous example of an adult living organism Hydra [3] are illustrations of spectacular spontaneous cell sorting. Steinberg [4-7] used the ability of cells to self-organize in coherent structures to conduct a series of pioneering experimental studies that characterized cell adhesion as a major actor of cell sorting. Following his experiments, Steinberg suggested that the interaction between two cells involves an adhesion surface energy which varies according to the cell type. To interpret cell sorting, Steinberg formulated the Differential Adhesion Hypothesis (DAH), which states that cells can explore various configurations and finally reach the lowest-energy configuration. During the past decades, the DAH has been experimentally tested in various situations such as gastrulation [8], cell shaping [9], control of pattern formation [10] and the engulfment of a tissue by another one. Some of these experiments have been recently improved to support the DAH with more evidence [11].

In the 80's and the 90's, the DAH inspired the development of many mathematical models. These models, recently reviewed in [12], rely on computer simulations of physical processes. In summary, these models act by minimizing an energy functional called the Hamiltonian, and they can be classified into four main groups according to the geometry chosen to describe the tissues.

First, *cell-lattice models *consider that each cell is geometrically described by a common shape, generally a regular polygon (square, hexagon, etc ...) (see [13] for example). Although these models may not be realistic due to the simple representation of each cell, their computation is straightforward and fast. The second class of models has been called *centric models*. In comparison with the cell-lattice models, centric models are based on more realistic cell geometries by using tessellations to define cell boundaries from a point pattern where points characterize cell centers [14]. While the main benefit of this class of models is the use of a continuous geometry, tessellation algorithms are known to be computationally slow [12]. The third class of models are the *vertex models*. These models are dual to the centric models [15,16], and they have the same characteristics in terms of realism and computational behavior. The fourth class of models, called *sub-cellular lattice models*, has been developed as a trade-off between the simulation speed of cell-lattice models and the geometrical flexibility of the centric models. The first sub-cellular lattice model was introduced by Graner and Glazier (*GG model*) [17].

Tuning the internal parameters of centric or lattice models is usually achieved by direct comparison of the model output and the real data that they are supposed to mimic. An important challenge is to provide automatic estimation procedures for these parameters based on statistically consistent models and algorithms. For example, it is now acknowledged that cell-cell interactions play a major role in tumorigenesis [18]. Better understanding and estimating the nature of these interactions may play a key role for an early detection of cancer. In addition, the invasive nature of some tumors is directly linked to the modification of the strength of cell-cell interactions [19]. Estimating this parameter could therefore be a step toward more accurate prognosis.

In this study, we present a statistical approach to the estimation of the strength of adhesion between cells under the DAH, based on a continuous stochastic model for cell sorting rather than a discrete one. Our model is inspired by the pioneering works of Sulsky *et al*. [20], Graner and Sawada (GS model) [21] and from the GG model [17]. In the new model, the geometry of cells is actually similar to the centric models: assuming that cell centers are known, the cells are approximated by Dirichlet cells. Using the theory of Gibbsian marked point processes [22], the continuous model can still be described through a Hamiltonian function (Section "A continuous model for DAH"). The Gibbsian marked point processes theory contains standard procedures to estimate interaction parameters. In addition, it allows us to provide more rigorous simulation algorithms including better control of mixing properties, and it also provides a tool for establish consistency of estimators (Section "Inference procedure and model simulation"). In Section "Results and Discussion", results concerning the simulation of classical cell sorting patterns using this new model are reported, and the performances of the cell-cell adhesion strength estimator derived from this model are evaluated.

## A continuous model for DAH

In this section, a new continuous model for differential adhesion is introduced. Like previous approaches, the model is based on a Hamiltonian function that describes cell-cell interactions. The Hamiltonian function incorporates two terms: an interaction term and a shape constraint term. The interaction term refers to the DAH through a differential expression of Cellular Adhesion Molecules (CAMs) weighted by the length of the membrane separating cells. This model is inspired by cell-cell interactions driven by cadherin-catenin complexes [23] which are known to be implicated in cancerous processes [24]. The main characteristic of interactions driven by cadherin-catenin complexes is that the strength of adhesion is proportional to the length of the membrane shared by two contiguous cells. This particularity is due to a zipper-like crystalline structure of cadherin interactions [25]. The constraint term relates to the shape of biological cells and prevent non-realistic cell shapes.

The proposed model uses a Dirichlet tessellation as a representation of cell geometry. The Dirichlet tessellation is entirely specified from the locations of the cell centers. Formally, we denote by *x*_*i *_(*i *= 1, ..., *n*) the *n *cell centers, where *x*_*i *_is assumed to belong to a non-empty compact subset X
 MathType@MTEF@5@5@+=feaafiart1ev1aaatCvAUfKttLearuWrP9MDH5MBPbIqV92AaeXatLxBI9gBaebbnrfifHhDYfgasaacH8akY=wiFfYdH8Gipec8Eeeu0xXdbba9frFj0=OqFfea0dXdd9vqai=hGuQ8kuc9pgc9s8qqaq=dirpe0xb9q8qiLsFr0=vr0=vr0dc8meaabaqaciaacaGaaeqabaqabeGadaaakeGabaaBymrtHrhAL1wy0L2yHvtyaeHbnfgDOvwBHrxAJfwnaGabaiab=Dr8ybaa@38D5@ of ℝ^2^. The Dirichlet cell of *x*_*i *_is denoted by Dir(*x*_*i*_), and is defined as the set of points (within X
 MathType@MTEF@5@5@+=feaafiart1ev1aaatCvAUfKttLearuWrP9MDH5MBPbIqV92AaeXatLxBI9gBaebbnrfifHhDYfgasaacH8akY=wiFfYdH8Gipec8Eeeu0xXdbba9frFj0=OqFfea0dXdd9vqai=hGuQ8kuc9pgc9s8qqaq=dirpe0xb9q8qiLsFr0=vr0=vr0dc8meaabaqaciaacaGaaeqabaqabeGadaaakeGabaaBymrtHrhAL1wy0L2yHvtyaeHbnfgDOvwBHrxAJfwnaGabaiab=Dr8ybaa@38D5@) which are closer to *x*_*i *_than to any other cell centers. Let us denote a (marked) cell configuration as

*ϕ *= {(*x*_1_, *τ*_1_), ..., (*x*_*n*_, *τ*_*n*_)},

where the (*x*_*i*_) are the cell centers, and the (*τ*_*i*_) are the corresponding cell types (or marks). The marks belong to a finite discrete space *M*. In the section "Results and Discussion", we consider the case where cells may be of one of the three types: *M *= {*τ*_1_, *τ*_2_, *τ*_*E*_}, in analogy with cell types used in [26].

The interaction term corresponds to pair potentials and it controls the adhesion forces between contiguous cells. This term is defined as follows

Hinter(ϕ)=∑i~ϕj|Dir(xi∩xj)|J(τi,τj)
 MathType@MTEF@5@5@+=feaafiart1ev1aaatCvAUfKttLearuWrP9MDH5MBPbIqV92AaeXatLxBI9gBaebbnrfifHhDYfgasaacH8akY=wiFfYdH8Gipec8Eeeu0xXdbba9frFj0=OqFfea0dXdd9vqai=hGuQ8kuc9pgc9s8qqaq=dirpe0xb9q8qiLsFr0=vr0=vr0dc8meaabaqaciaacaGaaeqabaqabeGadaaakeaacqWGibasdaWgaaWcbaGaeeyAaKMaeeOBa4MaeeiDaqNaeeyzauMaeeOCaihabeaakiabcIcaOGGaciab=v9aQjabcMcaPiabg2da9maaqafabaWaaqWaaeaacqqGebarcqqGPbqAcqqGYbGCcqGGOaakcqWG4baEdaWgaaWcbaGaemyAaKgabeaakiabgMIihlabdIha4naaBaaaleaacqWGQbGAaeqaaOGaeiykaKcacaGLhWUaayjcSdGaemOsaOKaeiikaGIae8hXdq3aaSbaaSqaaiabdMgaPbqabaGccqGGSaalcqWFepaDdaWgaaWcbaGaemOAaOgabeaakiabcMcaPaWcbaGaemyAaKMaeiOFa43aaSbaaWqaaiab=v9aQbqabaWccqWGQbGAaeqaniabggHiLdaaaa@5C79@

where |Dir(*x*_*i *_∩ *x*_*j*_)| denotes the length of the contact zone between cell *x*_*i *_and cell *x*_*j*_. Function *J *is assumed to be symmetric and nonnegative

*J *: *M *× *M *→ [0, ∞)

The symbol *i *~_*ϕ *_*j *means that the cells *x*_*i *_and *x*_*j *_share a common edge in the Dirichlet tiling built from the configuration of points in *ϕ*.

The shape constraint term corresponds to singleton potentials. It controls the form of each cells and puts a penalty on abnormally large cells. It is defined as follows

Hshape(ϕ)=∑ih(Dir(xi),τi)
 MathType@MTEF@5@5@+=feaafiart1ev1aaatCvAUfKttLearuWrP9MDH5MBPbIqV92AaeXatLxBI9gBaebbnrfifHhDYfgasaacH8akY=wiFfYdH8Gipec8Eeeu0xXdbba9frFj0=OqFfea0dXdd9vqai=hGuQ8kuc9pgc9s8qqaq=dirpe0xb9q8qiLsFr0=vr0=vr0dc8meaabaqaciaacaGaaeqabaqabeGadaaakeaacqWGibasdaWgaaWcbaGaee4CamNaeeiAaGMaeeyyaeMaeeiCaaNaeeyzaugabeaakiabcIcaOGGaciab=v9aQjabcMcaPiabg2da9maaqafabaGaemiAaGMaeiikaGIaeeiraqKaeeyAaKMaeeOCaiNaeiikaGIaemiEaG3aaSbaaSqaaiabdMgaPbqabaGccqGGPaqkcqGGSaalcqWFepaDdaWgaaWcbaGaemyAaKgabeaakiabcMcaPaWcbaGaemyAaKgabeqdcqGHris5aaaa@4C94@

where the function *h *is assumed to be nonnegative

*h *: D
 MathType@MTEF@5@5@+=feaafiart1ev1aaatCvAUfKttLearuWrP9MDH5MBPbIqV92AaeXatLxBI9gBaebbnrfifHhDYfgasaacH8akY=wiFfYdH8Gipec8Eeeu0xXdbba9frFj0=OqFfea0dXdd9vqai=hGuQ8kuc9pgc9s8qqaq=dirpe0xb9q8qiLsFr0=vr0=vr0dc8meaabaqaciaacaGaaeqabaqabeGadaaakeGabaaBymrtHrhAL1wy0L2yHvtyaeHbnfgDOvwBHrxAJfwnaGabaiab=nq8ebaa@38AD@ × *M *→ [0, ∞)

One specific form of the term *h*(Dir(*x*_*i*_), *τ*_*i*_), used as an example in this paper, will be described in the section "Results and Discussion". The energy functional of our model is defined as a combination of the interaction term and the shape constraint as follows

*H*(*ϕ*) = *θ H*_inter _(*ϕ*) + *H*_shape _(*ϕ*)

where *θ *is a positive parameter. This parameter can be interpreted as an adhesion strength intensity, as it determines the relative contribution of cell-cell interactions in the energy. It may reflect the general state of a tissue, and its inference is relevant to applications of the model to experimental data.

Since one considers finite configurations *ϕ *on the compact set X
 MathType@MTEF@5@5@+=feaafiart1ev1aaatCvAUfKttLearuWrP9MDH5MBPbIqV92AaeXatLxBI9gBaebbnrfifHhDYfgasaacH8akY=wiFfYdH8Gipec8Eeeu0xXdbba9frFj0=OqFfea0dXdd9vqai=hGuQ8kuc9pgc9s8qqaq=dirpe0xb9q8qiLsFr0=vr0=vr0dc8meaabaqaciaacaGaaeqabaqabeGadaaakeGabaaBymrtHrhAL1wy0L2yHvtyaeHbnfgDOvwBHrxAJfwnaGabaiab=Dr8ybaa@38D5@ × ℳ
 MathType@MTEF@5@5@+=feaafiart1ev1aaatCvAUfKttLearuWrP9MDH5MBPbIqV92AaeXatLxBI9gBaebbnrfifHhDYfgasaacH8akY=wiFfYdH8Gipec8Eeeu0xXdbba9frFj0=OqFfea0dXdd9vqai=hGuQ8kuc9pgc9s8qqaq=dirpe0xb9q8qiLsFr0=vr0=vr0dc8meaabaqaciaacaGaaeqabaqabeGadaaakeGabaaBymrtHrhAL1wy0L2yHvtyaeHbnfgDOvwBHrxAJfwnaGabaiab=ntinbaa@3816@, the energy functional *H*(*ϕ*) is finite (|*H*(*ϕ*)| < ∞). Indeed, one can notice that the area of the cell |Dir(*x*_*i*_)| is bounded by the area of the compact set X
 MathType@MTEF@5@5@+=feaafiart1ev1aaatCvAUfKttLearuWrP9MDH5MBPbIqV92AaeXatLxBI9gBaebbnrfifHhDYfgasaacH8akY=wiFfYdH8Gipec8Eeeu0xXdbba9frFj0=OqFfea0dXdd9vqai=hGuQ8kuc9pgc9s8qqaq=dirpe0xb9q8qiLsFr0=vr0=vr0dc8meaabaqaciaacaGaaeqabaqabeGadaaakeGabaaBymrtHrhAL1wy0L2yHvtyaeHbnfgDOvwBHrxAJfwnaGabaiab=Dr8ybaa@38D5@. Coupling with the fact that *h *is a real-valued function, it comes that *H*_shape _is bounded. Similarly, the length of a common edge |Dir(*x*_*i *_∩ *x*_*j*_)| is bounded by the diameter of the compact set X
 MathType@MTEF@5@5@+=feaafiart1ev1aaatCvAUfKttLearuWrP9MDH5MBPbIqV92AaeXatLxBI9gBaebbnrfifHhDYfgasaacH8akY=wiFfYdH8Gipec8Eeeu0xXdbba9frFj0=OqFfea0dXdd9vqai=hGuQ8kuc9pgc9s8qqaq=dirpe0xb9q8qiLsFr0=vr0=vr0dc8meaabaqaciaacaGaaeqabaqabeGadaaakeGabaaBymrtHrhAL1wy0L2yHvtyaeHbnfgDOvwBHrxAJfwnaGabaiab=Dr8ybaa@38D5@, and providing that *J *is a real-valued function, *H*_inter _is also bounded. Moreover, since *J *and *h *are positive functions and *θ *> 0, *H*(*ϕ*) is even positive.

Before giving an inference procedure for *θ*, we describe the connections of our continuous model to earlier models, for which no such procedure exists. The new continuous model improves on three previous approaches by Sulsky *et al*. [20], Graner and Sawada [21] and Graner and Glazier [17]. Sulsky *et al*. proposed a model of cell sorting [20] according to a parallel between cell movements and fluid dynamics. A Dirichlet tessellation was used for modeling cells, the following Hamiltonian was introduced

HS=∑i~j|Dir(xi∩xj)|ei,j
 MathType@MTEF@5@5@+=feaafiart1ev1aaatCvAUfKttLearuWrP9MDH5MBPbIqV92AaeXatLxBI9gBaebbnrfifHhDYfgasaacH8akY=wiFfYdH8Gipec8Eeeu0xXdbba9frFj0=OqFfea0dXdd9vqai=hGuQ8kuc9pgc9s8qqaq=dirpe0xb9q8qiLsFr0=vr0=vr0dc8meaabaqaciaacaGaaeqabaqabeGadaaakeaaieGacqWFibasdaWgaaWcbaGaee4uamfabeaakiabg2da9maaqafabaWaaqWaaeaacqqGebarcqqGPbqAcqqGYbGCcqGGOaakcqWG4baEdaWgaaWcbaGaemyAaKgabeaakiabgMIihlabdIha4naaBaaaleaacqWGQbGAaeqaaOGaeiykaKcacaGLhWUaayjcSdGaemyzau2aaSbaaSqaaiabdMgaPjabcYcaSiabdQgaQbqabaaabaGaemyAaKMaeiOFa4NaemOAaOgabeqdcqGHris5aaaa@4BFB@

where *e*_*i*, *j *_is the interaction energy between cells *x*_*i *_and *x*_*j*_. As in our new continuous model, the length of the membrane also contributes to the energy. Graner and Sawada described another geometrical model for cell rearrangement [21]. Graner and Sawada introduced "free Dirichlet domains", which are an extension of Dirichlet domains, to overcome the excess of regular shapes in classical Dirichlet tilings. In addition to this geometrical representation, Graner and Sawada proposed an extension to Sulsky's Hamiltonian accounting for the interaction between cells and the external medium

HGS=∑i~j|Dir(xi∩xj)|ei,j+∑i|Dir(xi∩M)|ei,M
 MathType@MTEF@5@5@+=feaafiart1ev1aaatCvAUfKttLearuWrP9MDH5MBPbIqV92AaeXatLxBI9gBaebbnrfifHhDYfgasaacH8akY=wiFfYdH8Gipec8Eeeu0xXdbba9frFj0=OqFfea0dXdd9vqai=hGuQ8kuc9pgc9s8qqaq=dirpe0xb9q8qiLsFr0=vr0=vr0dc8meaabaqaciaacaGaaeqabaqabeGadaaakeaacqWGibasdaWgaaWcbaGaee4raCKaee4uamfabeaakiabg2da9maaqafabaWaaqWaaeaacqqGebarcqqGPbqAcqqGYbGCcqGGOaakcqWG4baEdaWgaaWcbaGaemyAaKgabeaakiabgMIihlabdIha4naaBaaaleaacqWGQbGAaeqaaOGaeiykaKcacaGLhWUaayjcSdGaemyzau2aaSbaaSqaaiabdMgaPjabcYcaSiabdQgaQbqabaaabaGaemyAaKMaeiOFa4NaemOAaOgabeqdcqGHris5aOGaey4kaSYaaabuaeaadaabdaqaaiabbseaejabbMgaPjabbkhaYjabcIcaOiabdIha4naaBaaaleaacqWGPbqAaeqaaOGaeyykICSaemyta0KaeiykaKcacaGLhWUaayjcSdGaemyzau2aaSbaaSqaaiabdMgaPjabcYcaSiabd2eanbqabaaabaGaemyAaKgabeqdcqGHris5aaaa@64B7@

where |Dir(*x*_*i *_∩ *M*)| is the length of the membrane between cell *x*_*i *_and the extracellular medium. This term is equal to 0 if the extracellular medium is not in the neighbourhood of *x*_*i*_. While the length of the membrane is explicitly included in the models, no statistical estimate for the interaction strength was proposed in these two approaches.

In the GG model [17], a cell is not defined as a simple unit, but instead consists of several pixels. The cells can belong to three types: high surface energy cells, low surface energy cells or medium cells, which were coded as 1, 2 and -1 in the original approach. According to the DAH, Hamiltonian *H*_GG _was defined as follows

HGG=∑(i,j)~(i′,j′)J(τ(σij),τ(σi′j′))(1−δσij,σi′j′)+λ∑σ(a(σ)−Aτ(σ))2Γ(Aτ(σ)),
 MathType@MTEF@5@5@+=feaafiart1ev1aaatCvAUfKttLearuWrP9MDH5MBPbIqV92AaeXatLxBI9gBaebbnrfifHhDYfgasaacH8akY=wiFfYdH8Gipec8Eeeu0xXdbba9frFj0=OqFfea0dXdd9vqai=hGuQ8kuc9pgc9s8qqaq=dirpe0xb9q8qiLsFr0=vr0=vr0dc8meaabaqaciaacaGaaeqabaqabeGadaaakeaacqWGibasdaWgaaWcbaGaee4raCKaee4raCeabeaakiabg2da9maaqafabaGaemOsaOKaeiikaGccciGae8hXdqNaeiikaGIae83Wdm3aaSbaaSqaaiabdMgaPjabdQgaQbqabaGccqGGPaqkcqGGSaalcqWFepaDcqGGOaakcqWFdpWCdaWgaaWcbaGafmyAaKMbauaacuWGQbGAgaqbaaqabaGccqGGPaqkcqGGPaqkaSqaaiabcIcaOiabdMgaPjabcYcaSiabdQgaQjabcMcaPiabc6ha+jabcIcaOiqbdMgaPzaafaGaeiilaWIafmOAaOMbauaacqGGPaqkaeqaniabggHiLdGcdaqadaqaaiabigdaXiabgkHiTiab=r7aKnaaBaaaleaacqWFdpWCdaWgaaadbaGaemyAaKMaemOAaOgabeaaliabcYcaSiab=n8aZnaaBaaameaacuWGPbqAgaqbaiqbdQgaQzaafaaabeaaaSqabaaakiaawIcacaGLPaaacqGHRaWkcqWF7oaBdaaeqbqaaiabcIcaOiabdggaHjabcIcaOiab=n8aZjabcMcaPiabgkHiTiabdgeabnaaBaaaleaacqWFepaDcqGGOaakcqWFdpWCcqGGPaqkaeqaaOGaeiykaKYaaWbaaSqabeaacqaIYaGmaaGccqqHtoWrcqGGOaakcqWGbbqqdaWgaaWcbaGae8hXdqNaeiikaGIae83WdmNaeiykaKcabeaakiabcMcaPiabcYcaSaWcbaGae83WdmhabeqdcqGHris5aaaa@82F8@

where (*i, j*) are the pixel spatial coordinates, *σ*_*ij *_represents the cell to which the pixel (*i, j*) belongs, *τ*(*σ*_*ij*_) denotes the type of the cell *σ*_*ij*_, and the function *J *characterizes the interaction intensity between two cell types (*δ *denoted the Kronecker symbol). The neigbourhood relationship used by Graner and Glazier is of second order which means that diagonal pixels interact. The term (1−δσij,σi′j′)
 MathType@MTEF@5@5@+=feaafiart1ev1aaatCvAUfKttLearuWrP9MDH5MBPbIqV92AaeXatLxBI9gBaebbnrfifHhDYfgasaacH8akY=wiFfYdH8Gipec8Eeeu0xXdbba9frFj0=OqFfea0dXdd9vqai=hGuQ8kuc9pgc9s8qqaq=dirpe0xb9q8qiLsFr0=vr0=vr0dc8meaabaqaciaacaGaaeqabaqabeGadaaakeaadaqadaqaaiabigdaXiabgkHiTGGaciab=r7aKnaaBaaaleaacqWFdpWCdaWgaaadbaGaemyAaKMaemOAaOgabeaaliabcYcaSiab=n8aZnaaBaaameaacuWGPbqAgaqbaiqbdQgaQzaafaaabeaaaSqabaaakiaawIcacaGLPaaaaaa@3C48@ indicates that the interaction between two pixels within the same cell is zero. Shape constraints are modeled by the second term where *λ *corresponds to an elasticity coefficient, *a*(*σ*) is the cell area and *A*_*τ *(*σ*) _is a prior area of a cell of type *τ *> 0. The function Γ denotes the Heaviside function and is included in the formula so that medium cells (coding -1) are not subject to the shape constraint. This model is simulated using the Boltzmann dynamics with various parameter settings and is able to reproduce many biologically relevant patterns [26]. The model introduced in this paper is a formal extension of the continuous version of the GG model [17] and also of the models introduced by Sulsky *et al*. [20] and Graner and Sawada [21]. Let us now explain in which sense this extension works. In the GG model, a cell *σ *is in the neighbourhood of a cell *σ*' as soon as a single pixel of *σ *is adjacent to a pixel from *σ*'. With this in mind, the GG model's Hamiltonian can be rewritten as

HGG=∑σ~σ′|σ∩σ′|J(τ(σ),τ(σ′))+λ∑σ(a(σ)−Aτ(σ))2Γ(Aτ(σ))
 MathType@MTEF@5@5@+=feaafiart1ev1aaatCvAUfKttLearuWrP9MDH5MBPbIqV92AaeXatLxBI9gBaebbnrfifHhDYfgasaacH8akY=wiFfYdH8Gipec8Eeeu0xXdbba9frFj0=OqFfea0dXdd9vqai=hGuQ8kuc9pgc9s8qqaq=dirpe0xb9q8qiLsFr0=vr0=vr0dc8meaabaqaciaacaGaaeqabaqabeGadaaakeaacqWGibasdaWgaaWcbaGaee4raCKaee4raCeabeaakiabg2da9maaqafabaWaaqWaaeaaiiGacqWFdpWCcqGHPiYXcuWFdpWCgaqbaaGaay5bSlaawIa7aiabdQeakjabcIcaOiab=r8a0jabcIcaOiab=n8aZjabcMcaPiabcYcaSiab=r8a0jabcIcaOiqb=n8aZzaafaGaeiykaKIaeiykaKcaleaacqWFdpWCcqGG+bGFcuWFdpWCgaqbaaqab0GaeyyeIuoakiabgUcaRiab=T7aSnaaqafabaGaeiikaGIaemyyaeMaeiikaGIae83WdmNaeiykaKIaeyOeI0Iaemyqae0aaSbaaSqaaiab=r8a0jabcIcaOiab=n8aZjabcMcaPaqabaGccqGGPaqkdaahaaWcbeqaaiabikdaYaaakiabfo5ahjabcIcaOiabdgeabnaaBaaaleaacqWFepaDcqGGOaakcqWFdpWCcqGGPaqkaeqaaOGaeiykaKcaleaacqWFdpWCaeqaniabggHiLdaaaa@6DC4@

where |*σ *∩ *σ*'| is the number of connected pixels between *σ *and *σ*'. The quantity |*σ *∩ *σ*'| can be identified as the Euclidean length of the interaction surface between the two cells *σ *and *σ*'. Identifying cells to their centers, |*σ *∩ *σ*'| can be approximated as |Dir(*x*_*i *_∩ *x*_*j*_)|. In addition, a cell area in our model matches with the area of a Dirichlet cell, which means that *a*(*σ*) corresponds to |Dir(*x*_*i*_)|. Using these notations, the GG energy function can be rewritten in a form similar to our Hamiltonian

H(ϕ)=∑i~ϕj|Dir(xi∩xj)|J(τi,τj)+λ∑i(|Dir(xi)|−Aτi)2Γ(Aτi).
 MathType@MTEF@5@5@+=feaafiart1ev1aaatCvAUfKttLearuWrP9MDH5MBPbIqV92AaeXatLxBI9gBaebbnrfifHhDYfgasaacH8akY=wiFfYdH8Gipec8Eeeu0xXdbba9frFj0=OqFfea0dXdd9vqai=hGuQ8kuc9pgc9s8qqaq=dirpe0xb9q8qiLsFr0=vr0=vr0dc8meaabaqaciaacaGaaeqabaqabeGadaaakeaacqWGibascqGGOaakiiGacqWFvpGAcqGGPaqkcqGH9aqpdaaeqbqaamaaemaabaGaeeiraqKaeeyAaKMaeeOCaiNaeiikaGIaemiEaG3aaSbaaSqaaiabdMgaPbqabaGccqGHPiYXcqWG4baEdaWgaaWcbaGaemOAaOgabeaakiabcMcaPaGaay5bSlaawIa7aiabdQeakjabcIcaOiab=r8a0naaBaaaleaacqWGPbqAaeqaaOGaeiilaWIae8hXdq3aaSbaaSqaaiabdQgaQbqabaGccqGGPaqkaSqaaiabdMgaPjabc6ha+naaBaaameaacqWFvpGAaeqaaSGaemOAaOgabeqdcqGHris5aOGaey4kaSIae83UdW2aaabuaeaacqGGOaakdaabdaqaaiabbseaejabbMgaPjabbkhaYjabcIcaOiabdIha4naaBaaaleaacqWGPbqAaeqaaOGaeiykaKcacaGLhWUaayjcSdGaeyOeI0Iaemyqae0aaSbaaSqaaiab=r8a0naaBaaameaacqWGPbqAaeqaaaWcbeaakiabcMcaPmaaCaaaleqabaGaeGOmaidaaOGaeu4KdCKaeiikaGIaemyqae0aaSbaaSqaaiab=r8a0naaBaaameaacqWGPbqAaeqaaaWcbeaakiabcMcaPiabc6caUaWcbaGaemyAaKgabeqdcqGHris5aaaa@7814@

The second term in Equation 5 is a particular case of the shape constraint term (see Equation 2) taking

h(Dir(xi),τi)=λ(|Dir(xi)|−Aτi)2Γ(Aτi)i=1…n.
 MathType@MTEF@5@5@+=feaafiart1ev1aaatCvAUfKttLearuWrP9MDH5MBPbIqV92AaeXatLxBI9gBaebbnrfifHhDYfgasaacH8akY=wiFfYdH8Gipec8Eeeu0xXdbba9frFj0=OqFfea0dXdd9vqai=hGuQ8kuc9pgc9s8qqaq=dirpe0xb9q8qiLsFr0=vr0=vr0dc8meaabaqaciaacaGaaeqabaqabeGadaaakeaafaqabeqacaaabaGaemiAaGMaeiikaGIaeeiraqKaeeyAaKMaeeOCaiNaeiikaGIaemiEaG3aaSbaaSqaaiabdMgaPbqabaGccqGGPaqkcqGGSaaliiGacqWFepaDdaWgaaWcbaGaemyAaKgabeaakiabcMcaPiabg2da9iab=T7aSjabcIcaOmaaemaabaGaeeiraqKaeeyAaKMaeeOCaiNaeiikaGIaemiEaG3aaSbaaSqaaiabdMgaPbqabaGccqGGPaqkaiaawEa7caGLiWoacqGHsislcqWGbbqqdaWgaaWcbaGae8hXdq3aaSbaaWqaaiabdMgaPbqabaaaleqaaOGaeiykaKYaaWbaaSqabeaacqaIYaGmaaGccqqHtoWrcqGGOaakcqWGbbqqdaWgaaWcbaGae8hXdq3aaSbaaWqaaiabdMgaPbqabaaaleqaaOGaeiykaKcabaGaemyAaKMaeyypa0JaeGymaeJaeSOjGSKaemOBa4gaaiabc6caUaaa@61BC@

To conclude this section, the new continuous model, introduced in this paper, unifies main features inspired from the three previous approaches. First, it borrows from Sulsky *et al*. the Dirichlet geometry for cells. Next it considers interactions between cells and surrounding medium as Graner and Sawada did. And finally it borrows from Graner and Glazier an additional constraint on the shape of cells. In addition, one strength of the new model is the introduction of a new parameter which quantifies adhesion within a tissue.

## Inference procedure and model simulation

An important benefit of the continuous approach is that it allows to develop consistent statistical estimation procedures for the adhesion strength parameter *θ*. To achieve this, we use the theory of Gibbsian marked point processes which provides a natural framework for parameter estimation (see [22,27]). Gibbsian models, according to the statistical physics terminology, have been introduced and largely studied in [28] or [29]. The idea of modeling cell configurations with point processes has been introduced in the literature by [30] and [22].

Given the energy functional defined in equation 2, we introduce a new marked point processes that have a density *f*, with respect to the homogeneous Poisson process of intensity 1 (as in [31], p360, l.12), of the following form

f(ϕ,θ)=exp⁡(−H(ϕ))Z(θ)
 MathType@MTEF@5@5@+=feaafiart1ev1aaatCvAUfKttLearuWrP9MDH5MBPbIqV92AaeXatLxBI9gBaebbnrfifHhDYfgasaacH8akY=wiFfYdH8Gipec8Eeeu0xXdbba9frFj0=OqFfea0dXdd9vqai=hGuQ8kuc9pgc9s8qqaq=dirpe0xb9q8qiLsFr0=vr0=vr0dc8meaabaqaciaacaGaaeqabaqabeGadaaakeaacqWGMbGzcqGGOaakiiGacqWFvpGAcqGGSaalcqWF4oqCcqGGPaqkcqGH9aqpdaWcaaqaaiGbcwgaLjabcIha4jabcchaWjabcIcaOiabgkHiTiabdIeaijabcIcaOiab=v9aQjabcMcaPiabcMcaPaqaaiabdQfaAjabcIcaOiab=H7aXjabcMcaPaaaaaa@4532@

where *Z*(*θ*) is the partition function, and *θ *is the parameter of interest. The probability measure for the marks is assumed to be uniform on the space of marks *M*. As noted in the previous section, our energy functional *H*(*ϕ*) is positive and bounded. Then *H*(*ϕ*) is stable in the sense of [28] (definition 3.2.1, p33). It follows that the proposed point process is well-defined as *Z*(*θ*) is bounded. A realization of such a process is called a configuration and is denoted as *ϕ *. When *ϕ *has exactly *n *points, we can write

*ϕ *= {(*x*_1_, *ϕ*_1_), ..., (*x*_*n*_, *ϕ*_*n*_)},

as in Equation 1. A cell-mark couple (*x*_*i*_, *τ*_*i*_) is then called *a point*. We can notice that the model proposed in this study belongs to the class of the *nearest-neighbour markov point processes *introduced by [32] (see Appendix 1).

In statistics, estimating *θ *is usually based on a maximum-likelihood approach. However, this approach cannot be used because the computation of the partition function is in general a very hard problem apart for very small *n*. Hence, as in [22], we resort to a classical approximation: the pseudo-likelihood method, first introduced by Besag in the context of the analysis of dirty pictures [33] (see also [34]). For any configuration *ϕ*, the pseudo-likelihood is defined as the product over all elements of *ϕ *of the following conditional probabilities

PL(ϕ,θ)=∏{xi,τi}∈ϕProb({xi,τi}|ϕ\{xi,τi},θ)
 MathType@MTEF@5@5@+=feaafiart1ev1aaatCvAUfKttLearuWrP9MDH5MBPbIqV92AaeXatLxBI9gBaebbnrfifHhDYfgasaacH8akY=wiFfYdH8Gipec8Eeeu0xXdbba9frFj0=OqFfea0dXdd9vqai=hGuQ8kuc9pgc9s8qqaq=dirpe0xb9q8qiLsFr0=vr0=vr0dc8meaabaqaciaacaGaaeqabaqabeGadaaakeaacqqGqbaucqqGmbatcqGGOaakiiGacqWFvpGAcqGGSaalcqWF4oqCcqGGPaqkcqGH9aqpdaqeqbqaaiabbcfaqjabbkhaYjabb+gaVjabbkgaIjabcIcaOiabcUha7jabdIha4naaBaaaleaacqWGPbqAaeqaaOGaeiilaWIae8hXdq3aaSbaaSqaaiabdMgaPbqabaGccqGG9bqFcqGG8baFcqWFvpGAcqGGCbaxdaWgaaWcbaGaei4EaSNaemiEaG3aaSbaaWqaaiabdMgaPbqabaWccqGGSaalcqWFepaDdaWgaaadbaGaemyAaKgabeaaliabc2ha9bqabaGccqGGSaalcqWF4oqCcqGGPaqkaSqaaiabcUha7jabdIha4naaBaaameaacqWGPbqAaeqaaSGaeiilaWIae8hXdq3aaSbaaWqaaiabdMgaPbqabaWccqGG9bqFcqGHiiIZcqWFvpGAaeqaniabg+Givdaaaa@6866@

In this formula, the conditional probability of observing {*x*_*i*_, *τ*_*i*_} at *x*_*i*_, given the configuration outside *x*_*i*_, can be described as

Prob({xi,τi}|ϕ\{xi,τi},θ)=exp⁡(−Hϕ({xi,τi},θ))∫X∑m∈Mexp⁡(−Hϕ\{xi,τi}∪{y,m}({y,m},θ))dy
 MathType@MTEF@5@5@+=feaafiart1ev1aaatCvAUfKttLearuWrP9MDH5MBPbIqV92AaeXatLxBI9gBaebbnrfifHhDYfgasaacH8akY=wiFfYdH8Gipec8Eeeu0xXdbba9frFj0=OqFfea0dXdd9vqai=hGuQ8kuc9pgc9s8qqaq=dirpe0xb9q8qiLsFr0=vr0=vr0dc8meaabaqaciaacaGaaeqabaqabeGadaaakeaacqqGqbaucqqGYbGCcqqGVbWBcqqGIbGycqGGOaakcqGG7bWEcqWG4baEdaWgaaWcbaGaemyAaKgabeaakiabcYcaSGGaciab=r8a0naaBaaaleaacqWGPbqAaeqaaOGaeiyFa0NaeiiFaWNae8x1dOMaeiixaW1aaSbaaSqaaiabcUha7jabdIha4naaBaaameaacqWGPbqAaeqaaSGaeiilaWIae8hXdq3aaSbaaWqaaiabdMgaPbqabaWccqGG9bqFaeqaaOGaeiilaWIae8hUdeNaeiykaKIaeyypa0ZaaSaaaeaacyGGLbqzcqGG4baEcqGGWbaCcqGGOaakcqGHsislcqWGibasdaWgaaWcbaGae8x1dOgabeaakiabcIcaOiabcUha7jabdIha4naaBaaaleaacqWGPbqAaeqaaOGaeiilaWIae8hXdq3aaSbaaSqaaiabdMgaPbqabaGccqGG9bqFcqGGSaalcqWF4oqCcqGGPaqkcqGGPaqkaeaadaWdraqaamaaqababaGagiyzauMaeiiEaGNaeiiCaaNaeiikaGIaeyOeI0IaemisaG0aaSbaaSqaaiab=v9aQjabcYfaCnaaBaaameaacqGG7bWEcqWG4baEdaWgaaqaaiabdMgaPbqabaGaeiilaWIae8hXdq3aaSbaaeaacqWGPbqAaeqaaiabc2ha9bqabaWccqGHQicYcqGG7bWEcqWG5bqEcqGGSaalcqWGTbqBcqGG9bqFaeqaaOGaeiikaGIaei4EaSNaemyEaKNaeiilaWIaemyBa0MaeiyFa0NaeiilaWIae8hUdeNaeiykaKIaeiykaKIaemizaqMaemyEaKhaleaacqWGTbqBcqGHiiIZcqWGnbqtaeqaniabggHiLdaaleGabaaBymrtHrhAL1wy0L2yHvtyaeHbnfgDOvwBHrxAJfwnaGabaiab+Dr8ybqab0Gaey4kIipaaaaaaa@A8AD@

where *M *corresponds to the set of the possible cell types (or marks), and where *H*_*ϕ *_({*x*_*i*_, *τ*_*i*_}) represents the contribution of the marked cell {*x*_*i*_, *τ*_*i*_} in the expression of the Hamiltonian *H*(*ϕ*), *i.e*.

Hϕ({xi,τi},θ)=θ∑j~ϕi|Dir(xi∩xj)|J(τi,τj)+h(Dir(xi),τi).
 MathType@MTEF@5@5@+=feaafiart1ev1aaatCvAUfKttLearuWrP9MDH5MBPbIqV92AaeXatLxBI9gBaebbnrfifHhDYfgasaacH8akY=wiFfYdH8Gipec8Eeeu0xXdbba9frFj0=OqFfea0dXdd9vqai=hGuQ8kuc9pgc9s8qqaq=dirpe0xb9q8qiLsFr0=vr0=vr0dc8meaabaqaciaacaGaaeqabaqabeGadaaakeaacqWGibasdaWgaaWcbaacciGae8x1dOgabeaakiabcIcaOiabcUha7jabdIha4naaBaaaleaacqWGPbqAaeqaaOGaeiilaWIae8hXdq3aaSbaaSqaaiabdMgaPbqabaGccqGG9bqFcqGGSaalcqWF4oqCcqGGPaqkcqGH9aqpcqWF4oqCdaaeqbqaamaaemaabaGaeeiraqKaeeyAaKMaeeOCaiNaeiikaGIaemiEaG3aaSbaaSqaaiabdMgaPbqabaGccqGHPiYXcqWG4baEdaWgaaWcbaGaemOAaOgabeaakiabcMcaPaGaay5bSlaawIa7aiabdQeakjabcIcaOiab=r8a0naaBaaaleaacqWGPbqAaeqaaOGaeiilaWIae8hXdq3aaSbaaSqaaiabdQgaQbqabaGccqGGPaqkcqGHRaWkcqWGObaAcqGGOaakcqqGebarcqqGPbqAcqqGYbGCcqGGOaakcqWG4baEdaWgaaWcbaGaemyAaKgabeaakiabcMcaPiabcYcaSiab=r8a0naaBaaaleaacqWGPbqAaeqaaOGaeiykaKcaleaacqWGQbGAcqGG+bGFdaWgaaadbaGae8x1dOgabeaaliabdMgaPbqab0GaeyyeIuoakiabc6caUaaa@75AA@

Taking the logarithm of the pseudo-likelihood leads to

LPL(ϕ,θ)=∑{xi,τi}∈ϕ(−Hϕ({xi,τi},θ)+log⁡(∫X∑m∈Mexp⁡(−Hϕ\{xi,τi}∪{y,m}({y,m},θ))dy)),
 MathType@MTEF@5@5@+=feaafiart1ev1aaatCvAUfKttLearuWrP9MDH5MBPbIqV92AaeXatLxBI9gBaebbnrfifHhDYfgasaacH8akY=wiFfYdH8Gipec8Eeeu0xXdbba9frFj0=OqFfea0dXdd9vqai=hGuQ8kuc9pgc9s8qqaq=dirpe0xb9q8qiLsFr0=vr0=vr0dc8meaabaqaciaacaGaaeqabaqabeGadaaakeaacqqGmbatcqqGqbaucqqGmbatcqGGOaakiiGacqWFvpGAcqGGSaalcqWF4oqCcqGGPaqkcqGH9aqpdaaeqbqaamaabmaabaGaeyOeI0IaemisaG0aaSbaaSqaaiab=v9aQbqabaGccqGGOaakcqGG7bWEcqWG4baEdaWgaaWcbaGaemyAaKgabeaakiabcYcaSiab=r8a0naaBaaaleaacqWGPbqAaeqaaOGaeiyFa0NaeiilaWIae8hUdeNaeiykaKIaey4kaSIagiiBaWMaei4Ba8Maei4zaC2aaeWaaeaadaWdraqaamaaqafabaGagiyzauMaeiiEaGNaeiiCaaNaeiikaGIaeyOeI0IaemisaG0aaSbaaSqaaiab=v9aQjabcYfaCnaaBaaameaacqGG7bWEcqWG4baEdaWgaaqaaiabdMgaPbqabaGaeiilaWIae8hXdq3aaSbaaeaacqWGPbqAaeqaaiabc2ha9jabgQIiilabcUha7jabdMha5jabcYcaSiabd2gaTjabc2ha9bqabaaaleqaaOGaeiikaGIaei4EaSNaemyEaKNaeiilaWIaemyBa0MaeiyFa0NaeiilaWIae8hUdeNaeiykaKIaeiykaKIaemizaqMaemyEaKhaleaacqWGTbqBcqGHiiIZcqWGnbqtaeqaniabggHiLdaaleGabaaBymrtHrhAL1wy0L2yHvtyaeHbnfgDOvwBHrxAJfwnaGabaiab+Dr8ybqab0Gaey4kIipaaOGaayjkaiaawMcaaaGaayjkaiaawMcaaaWcbaGaei4EaSNaemiEaG3aaSbaaWqaaiabdMgaPbqabaWccqGGSaalcqWFepaDdaWgaaadbaGaemyAaKgabeaaliabc2ha9jabgIGiolab=v9aQbqab0GaeyyeIuoakiabcYcaSaaa@A25D@

and maximizing LPL(*θ*) provides an estimate of *θ*, namely

θ^
 MathType@MTEF@5@5@+=feaafiart1ev1aaatCvAUfKttLearuWrP9MDH5MBPbIqV92AaeXatLxBI9gBaebbnrfifHhDYfgasaacH8akY=wiFfYdH8Gipec8Eeeu0xXdbba9frFj0=OqFfea0dXdd9vqai=hGuQ8kuc9pgc9s8qqaq=dirpe0xb9q8qiLsFr0=vr0=vr0dc8meaabaqaciaacaGaaeqabaqabeGadaaakeaaiiGacuWF4oqCgaqcaaaa@2E79@ (*ϕ*) = argmax_*θ *_LPL(*ϕ*, *θ*)

which can be computed using standard numerical techniques.

In order to evaluate both the statistical cell configurations according to the distribution of the Gibbsian marked point process and evaluate the statistical performances of the estimator θ^
 MathType@MTEF@5@5@+=feaafiart1ev1aaatCvAUfKttLearuWrP9MDH5MBPbIqV92AaeXatLxBI9gBaebbnrfifHhDYfgasaacH8akY=wiFfYdH8Gipec8Eeeu0xXdbba9frFj0=OqFfea0dXdd9vqai=hGuQ8kuc9pgc9s8qqaq=dirpe0xb9q8qiLsFr0=vr0=vr0dc8meaabaqaciaacaGaaeqabaqabeGadaaakeaaiiGacuWF4oqCgaqcaaaa@2E79@, an MCMC algorithm have been implemented. The algorithm differs from the GS and GG algorithms notably since these methods were time-dependent and account for the path from the initial to final state. We apply a Metropolis-Hastings algorithm for point processes as described in [31].

At each iteration, the algorithm randomly chooses between three operations: inserting a cell within the region X
 MathType@MTEF@5@5@+=feaafiart1ev1aaatCvAUfKttLearuWrP9MDH5MBPbIqV92AaeXatLxBI9gBaebbnrfifHhDYfgasaacH8akY=wiFfYdH8Gipec8Eeeu0xXdbba9frFj0=OqFfea0dXdd9vqai=hGuQ8kuc9pgc9s8qqaq=dirpe0xb9q8qiLsFr0=vr0=vr0dc8meaabaqaciaacaGaaeqabaqabeGadaaakeGabaaBymrtHrhAL1wy0L2yHvtyaeHbnfgDOvwBHrxAJfwnaGabaiab=Dr8ybaa@38D5@, deleting a cell or displacing a cell within X
 MathType@MTEF@5@5@+=feaafiart1ev1aaatCvAUfKttLearuWrP9MDH5MBPbIqV92AaeXatLxBI9gBaebbnrfifHhDYfgasaacH8akY=wiFfYdH8Gipec8Eeeu0xXdbba9frFj0=OqFfea0dXdd9vqai=hGuQ8kuc9pgc9s8qqaq=dirpe0xb9q8qiLsFr0=vr0=vr0dc8meaabaqaciaacaGaaeqabaqabeGadaaakeGabaaBymrtHrhAL1wy0L2yHvtyaeHbnfgDOvwBHrxAJfwnaGabaiab=Dr8ybaa@38D5@. One iteration is detailed in the appendix (Appendix 2). From Equation 7, one can remark that only the variation in the energy is needed to compute the acceptance probability. Insertion, deletion and displacement of a cell in the configuration has been implemented using local changes as described in [35] and [36].

A second kind of benefit carried out by the use of marked point processes is to provide theoretical conditions that warrant the convergence of the simulation algorithm.

**Proposition 1 ***Let *X
 MathType@MTEF@5@5@+=feaafiart1ev1aaatCvAUfKttLearuWrP9MDH5MBPbIqV92AaeXatLxBI9gBaebbnrfifHhDYfgasaacH8akY=wiFfYdH8Gipec8Eeeu0xXdbba9frFj0=OqFfea0dXdd9vqai=hGuQ8kuc9pgc9s8qqaq=dirpe0xb9q8qiLsFr0=vr0=vr0dc8meaabaqaciaacaGaaeqabaqabeGadaaakeGabaaBymrtHrhAL1wy0L2yHvtyaeHbnfgDOvwBHrxAJfwnaGabaiab=Dr8ybaa@38D5@*be a compact subset of *ℝ^2 ^*and M be a finite discrete space. Let ϕ be a point configuration*

*ϕ *= {(*x*_1_, *τ*_1_), ..., (*x*_*n*_, *τ*_*n*_)}

Let us consider a Gibbsian marked point process as defined in Equation 2, and

H(ϕ)=θ∑i~ϕj|Dir(xi∩xj)|J(τi,τj)+∑ih(Dir(xi),τi),
 MathType@MTEF@5@5@+=feaafiart1ev1aaatCvAUfKttLearuWrP9MDH5MBPbIqV92AaeXatLxBI9gBaebbnrfifHhDYfgasaacH8akY=wiFfYdH8Gipec8Eeeu0xXdbba9frFj0=OqFfea0dXdd9vqai=hGuQ8kuc9pgc9s8qqaq=dirpe0xb9q8qiLsFr0=vr0=vr0dc8meaabaqaciaacaGaaeqabaqabeGadaaakeaacqWGibascqGGOaakiiGacqWFvpGAcqGGPaqkcqGH9aqpcqWF4oqCdaaeqbqaamaaemaabaGaeeiraqKaeeyAaKMaeeOCaiNaeiikaGIaemiEaG3aaSbaaSqaaiabdMgaPbqabaGccqGHPiYXcqWG4baEdaWgaaWcbaGaemOAaOgabeaakiabcMcaPaGaay5bSlaawIa7aiabdQeakjabcIcaOiab=r8a0naaBaaaleaacqWGPbqAaeqaaOGaeiilaWIae8hXdq3aaSbaaSqaaiabdQgaQbqabaGccqGGPaqkaSqaaiabdMgaPjabc6ha+naaBaaameaacqWFvpGAaeqaaSGaemOAaOgabeqdcqGHris5aOGaey4kaSYaaabuaeaacqWGObaAcqGGOaakcqqGebarcqqGPbqAcqqGYbGCcqGGOaakcqWG4baEdaWgaaWcbaGaemyAaKgabeaakiabcMcaPiabcYcaSiab=r8a0naaBaaaleaacqWGPbqAaeqaaOGaeiykaKcaleaacqWGPbqAaeqaniabggHiLdGccqGGSaalaaa@6C2C@

*where J charaterizes the interaction intensity and h the constraint on the shape of cells*.

*Assuming that J and h are nonnegative real-valued functions, the Markov chain generated by the simulation algorithm of the continuous model (see Appendix 2) is ergodic*.

The proof of proposition 1 can be derived along the same lines as [31] (Section 4, p. 364). It can be sketched as follows. First, it is clear that the transition probabilities of the proposed algorithm satisfy Equations 3.5–3.9 in [31] (p. 361–362). Next, in order to ensure the irreducibility of the Markov chain, the density of the process has to be hereditary (Definition 3.1 in [31], p. 360). The *nearest-neighbour markov *property of our model ensures its hereditary. Then by adapting the proof of Corollary 2 in Tierney ([37], Section 3.1, p. 1713), it follows that the chain is ergodic.

## Results and Discussion

### Simulation of biological patterns

In this section, we report simulation results obtained with three marks *M *= {*τ*_1_, *τ*_2_, *τ*_*E*_}. We provide evidence that our model has the ability to reproduce at least three kinds of biologically observed patterns: checkerboard, cell sorting and engulfment. The constraint shape function *h *is borrowed from the GG model, and is is defined as in Equation 6. The parameter *λ *controls the intensity of the shape constraint. It also acts on the density of points within the studied region X
 MathType@MTEF@5@5@+=feaafiart1ev1aaatCvAUfKttLearuWrP9MDH5MBPbIqV92AaeXatLxBI9gBaebbnrfifHhDYfgasaacH8akY=wiFfYdH8Gipec8Eeeu0xXdbba9frFj0=OqFfea0dXdd9vqai=hGuQ8kuc9pgc9s8qqaq=dirpe0xb9q8qiLsFr0=vr0=vr0dc8meaabaqaciaacaGaaeqabaqabeGadaaakeGabaaBymrtHrhAL1wy0L2yHvtyaeHbnfgDOvwBHrxAJfwnaGabaiab=Dr8ybaa@38D5@. In the following of this paper we consider X
 MathType@MTEF@5@5@+=feaafiart1ev1aaatCvAUfKttLearuWrP9MDH5MBPbIqV92AaeXatLxBI9gBaebbnrfifHhDYfgasaacH8akY=wiFfYdH8Gipec8Eeeu0xXdbba9frFj0=OqFfea0dXdd9vqai=hGuQ8kuc9pgc9s8qqaq=dirpe0xb9q8qiLsFr0=vr0=vr0dc8meaabaqaciaacaGaaeqabaqabeGadaaakeGabaaBymrtHrhAL1wy0L2yHvtyaeHbnfgDOvwBHrxAJfwnaGabaiab=Dr8ybaa@38D5@ to be the unit disc and *λ *has been fixed to 10,000.

Biological tissue configurations are often interpreted in terms of surface tension parameters. For instance, checkerboard patterns are usually associated with negative surface tensions, whereas cell sorting patterns are associated with positive surface tensions [17]. When two distinct cell types are considered, the surface tension between cells with the distinct types can be defined as

γ12=J(τ1,τ2)−J(τ1,τ1)+J(τ2,τ2)2
 MathType@MTEF@5@5@+=feaafiart1ev1aaatCvAUfKttLearuWrP9MDH5MBPbIqV92AaeXatLxBI9gBaebbnrfifHhDYfgasaacH8akY=wiFfYdH8Gipec8Eeeu0xXdbba9frFj0=OqFfea0dXdd9vqai=hGuQ8kuc9pgc9s8qqaq=dirpe0xb9q8qiLsFr0=vr0=vr0dc8meaabaqaciaacaGaaeqabaqabeGadaaakeaaiiGacqWFZoWzdaWgaaWcbaGaeGymaeJaeGOmaidabeaakiabg2da9iabdQeakjabcIcaOiab=r8a0naaBaaaleaacqaIXaqmaeqaaOGaeiilaWIae8hXdq3aaSbaaSqaaiabikdaYaqabaGccqGGPaqkcqGHsisldaWcaaqaaiabdQeakjabcIcaOiab=r8a0naaBaaaleaacqaIXaqmaeqaaOGaeiilaWIae8hXdq3aaSbaaSqaaiabigdaXaqabaGccqGGPaqkcqGHRaWkcqWGkbGscqGGOaakcqWFepaDdaWgaaWcbaGaeGOmaidabeaakiabcYcaSiab=r8a0naaBaaaleaacqaIYaGmaeqaaOGaeiykaKcabaGaeGOmaidaaaaa@50C0@

The two marks *τ*_1 _and *τ*_2 _characterize "active cell types", as defined in [17], with distinct phenotypes responsible for the adhesion process. For example, phenotypes may represent different levels of expression of cadherins. In addition, active cells are surrounded by an extracellular medium modeled by cells of type *τ*_*E*_. One hundred cells of type *τ*_*E *_were uniformly placed on the frontier of the unit disc X
 MathType@MTEF@5@5@+=feaafiart1ev1aaatCvAUfKttLearuWrP9MDH5MBPbIqV92AaeXatLxBI9gBaebbnrfifHhDYfgasaacH8akY=wiFfYdH8Gipec8Eeeu0xXdbba9frFj0=OqFfea0dXdd9vqai=hGuQ8kuc9pgc9s8qqaq=dirpe0xb9q8qiLsFr0=vr0=vr0dc8meaabaqaciaacaGaaeqabaqabeGadaaakeGabaaBymrtHrhAL1wy0L2yHvtyaeHbnfgDOvwBHrxAJfwnaGabaiab=Dr8ybaa@38D5@.

**Figure 1:**
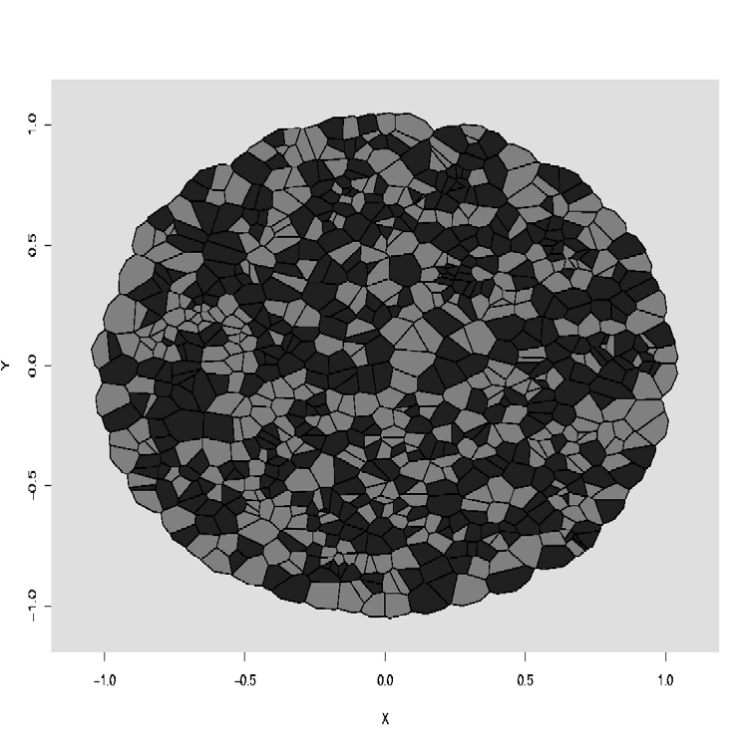
The initial configuration for simulating Checkerboard, Cell Sorting and Engulfment patterns. It consists of about 1,000 active cells surrounded by an extracellular medium. The active cells are randomly located in the unit sphere, and their types are randomly sampled from *M*. Cells of type *τ*_1 _are colored in black while cells of type *τ*_2 _are colored in grey. One hundred cells of type *τ*_*E *_were uniformely placed on the frontier of the unit disc.

These three types are similar to the ℓ, *d *and *M *types of Glazier and Graner [26]. Simulations were generated from the Metropolis algorithm presented in the previous section. A unique configuration was used to initialize all the simulations. This configuration is displayed in Figure 1. It consisted of about 1,000 uniformly located active cells, and the marks were also uniformly distributed in the mark space *M*. The target areas for active cells were equal to *A*_*τ*1 _= *A*_*τ*2 _= 5 × 10^-3^. At equilibrium, configurations were expected to consist of about *π*/5.10^-3 ^≈ 628 cells in the unit disc. No area constraint affected the *τ*_*E *_cells and we set *A*_*ϕ E *_= -1. The interaction term affecting two contiguous extracellular cells was set to the value *J*(*τ*_*E*_, *τ*_*E*_) = 0. The adhesion strength parameter *θ *was fixed to *θ *= 10.

**Figure 2:**
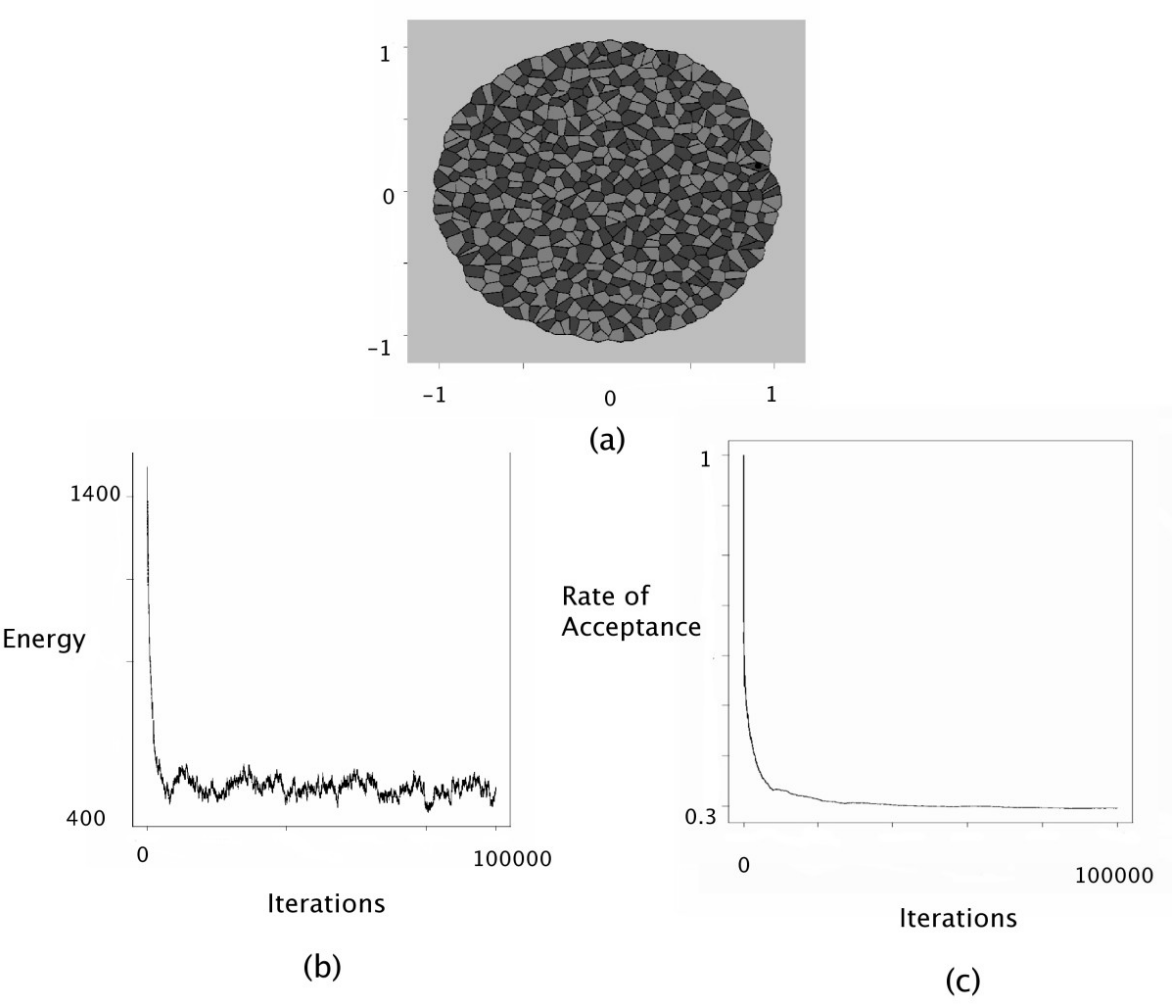
Checkerboard simulation. The interaction intensities were chosen as follows: *J*(*τ*_1_, *τ*_1_) = 1, *J*(*τ*_2_, *τ*_2_) = 1, *J*(*τ*_1_, *τ*_2_) = 0, *J*(*τ*_1_, *τ*_*E*_) = 0, *J*(*τ*_2_, *τ*_*E*_) = 0 and *J*(*τ*_*E*_, *τ*_*E*_) = 0. (a) The configuration obtained after 100,000 iterations with *θ *= 10. (b) The decrease of the energy as a function of the iteration steps. (c) The evolution of the accpetance rate as a function of the iteration steps.

Checkerboard patterns can be interpreted as arising from negative surface tensions. In the GG model, checkerboard patterns were generated using parameter settings that corresponded to a surface tension equal to *γ*_12 _= -3. Figure 2 displays the configuration obtained after 100,000 cycles of the Metropolis-Hastings algorithm, where the interaction intensities were fixed at *J*(*τ*_1_, *τ*_2_) = 0, *J*(*τ*_1_, *τ*_1_) = *J*(*τ*_2_, *τ*_2_) = 1 and *J*(*τ*_*E*_, *τ*_1_) = *J*(*τ*_*E*_, *τ*_2_) = 0. These interaction intensities correspond to a surface tension equal to *γ*_12 _= -1 which was of the same order as the one used in the GG model. Moreover we have *γ*_1*E *_= -1/2 and *γ*_2*E *_= -1/2.

**Figure 3:**
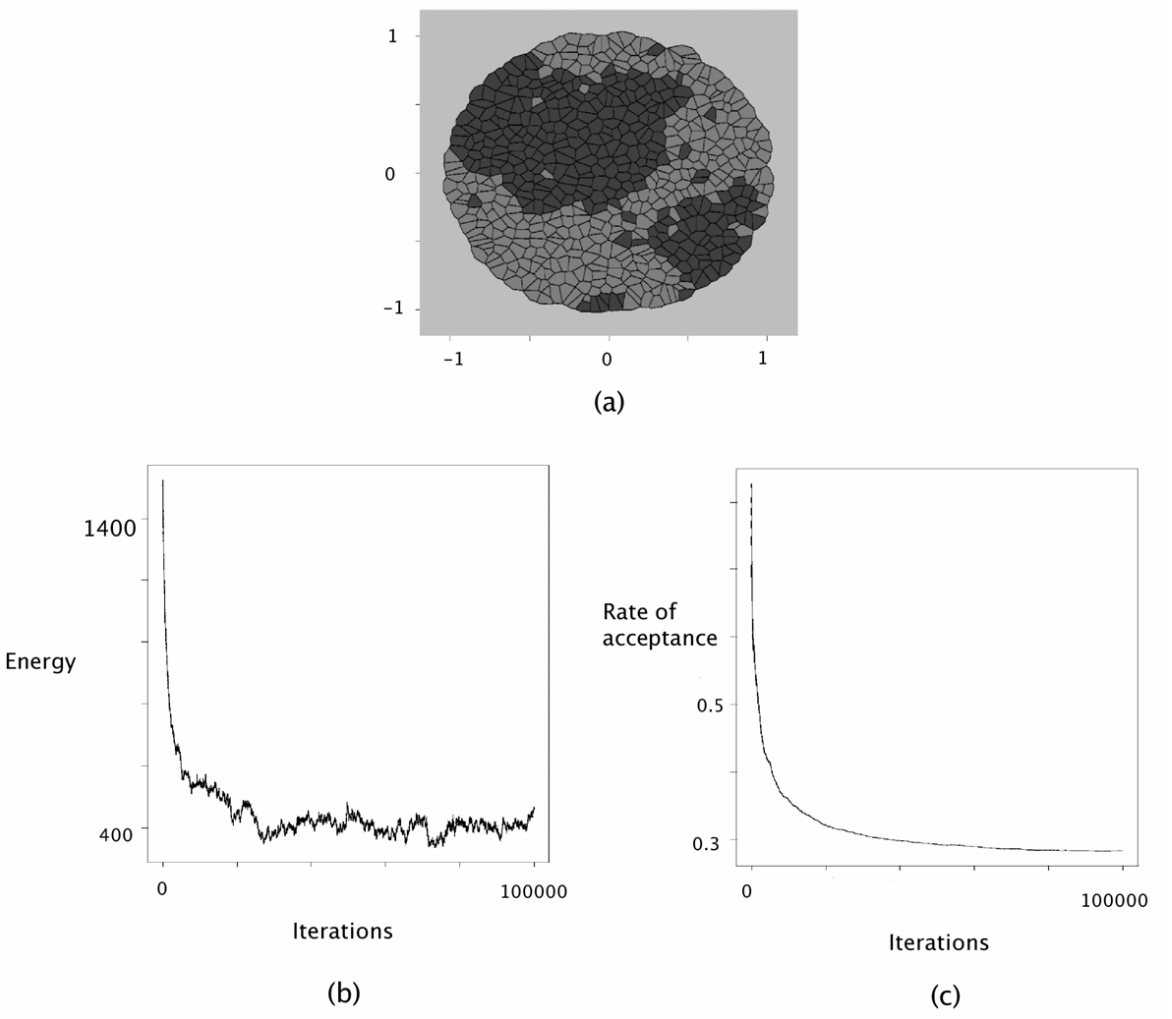
Cell Sorting simulation. The interaction intensities were chosen as follows: *J*(*τ*_1_, *τ*_1_) = 0, *J*(*τ*_2_, *τ*_2_) = 0, *J*(*τ*_1_, *τ*_2_) = 1, *J*(*τ*_1_, *τ*_*E*_) = 0, *J*(*τ*_2_, *τ*_*E*_) = 0 and *J*(*τ*_*E*_, *τ*_*E*_) = 0. (a) The configuration obtained after 100,000 iterations with *θ *= 10. (b) The decrease of the energy as a function of the iteration steps. (c) The evolution of the accpetance rate as a function of the iteration steps.

In contrast, cell sorting patterns arise from positive surface tensions between active cells. In the GG model, cell sorting patterns were generated using parameter settings that corresponded to surface tensions around *γ*_12 _= +3. In our model, simulations were conducted using the following interaction intensities:

*J*(*τ*_1_, *τ*_2_) = 1, *J*(*τ*_1_, *τ*_1_) = *J*(*τ*_2_, *τ*_2_) = 0 and *J*(*τ*_*E*_, *τ*_1_) = *J*(*τ*_*E*_, *τ*_2_) = 0. These values correspond to *γ*_12 _= +1. Surface tension with extracellular medium is equal to *γ*_1*E *_= 0 and *γ*_2*E *_= 0. The configuration obtained after 100,000 steps cycles of Metropolis-Hastings is displayed in Figure 3.

**Figure 4:**
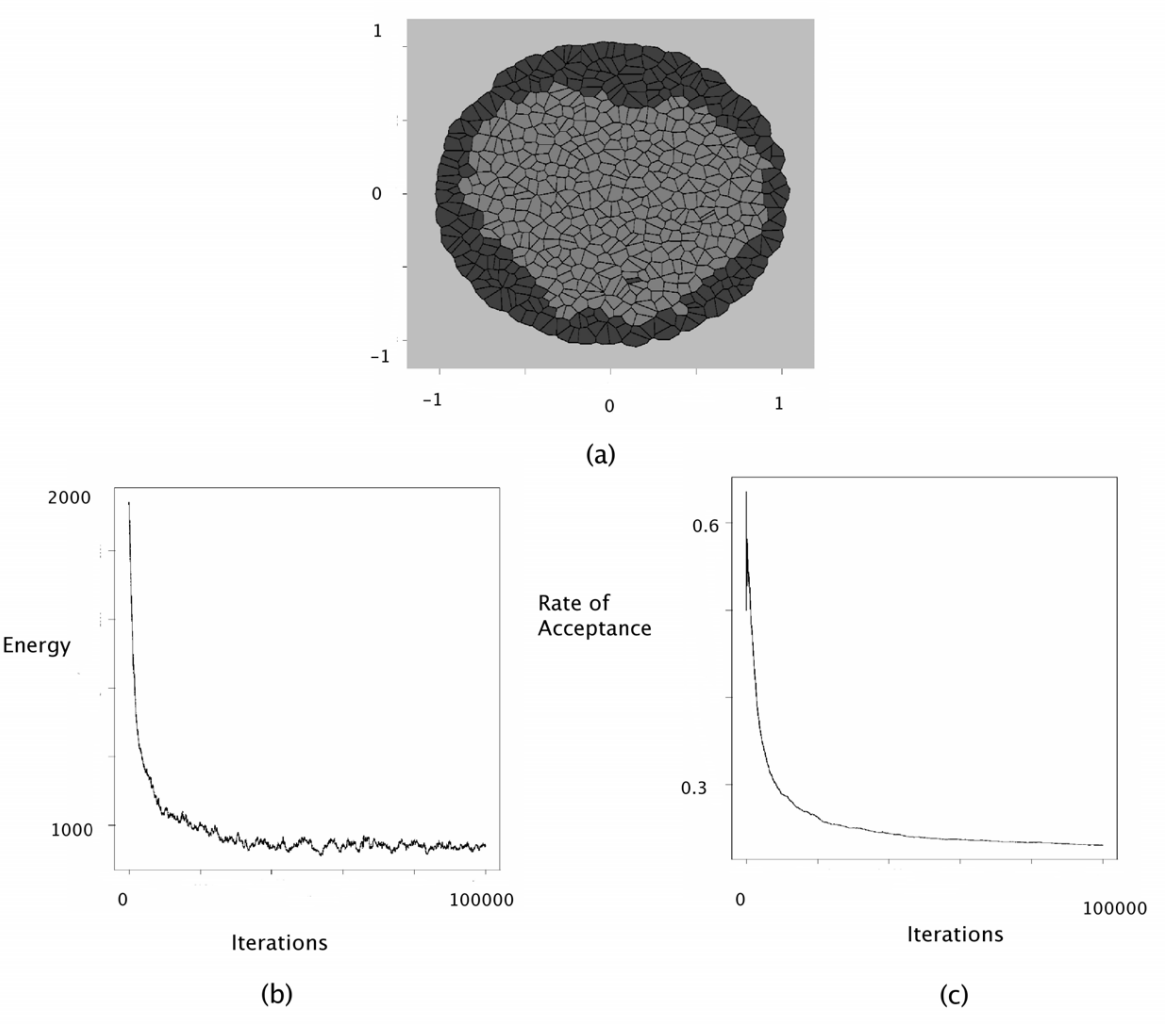
Engulfment simulation. The interaction intensities were chosen as follows: *J*(*τ*_1_, *τ*_1_) = 0, *J*(*τ*_2_, *τ*_2_) = 0, *J*(*τ*_1_, *τ*_2_) = 1, *J*(*τ*_1_, *τ*_*E*_) = 0, *J*(*τ*_2_, *τ*_*E*_) = 1 and *J*(*τ*_*E*_, *τ*_*E*_) = 1. (a) The configuration obtained after 100,000 iterations with *θ *= 10. (b) The decrease of the energy as a function of the iteration steps. (c) The evolution of the accpetance rate as a function of the iteration steps.

Simulations of engulfment were conducted using the following parameters: *J*(*τ*_1_, *τ*_2_) = 1, *J*(*τ*_1_, *τ*_1_) = *J*(*τ*_2_, *τ*_2_) = 0, *J*(*τ*_*E*_, *τ*_1_) = 0, *J*(*τ*_*E*_, *τ*_2_) = 1. These interaction intensities provide positive surface tensions between active cells, which contribute to the formation of clusters. The fact that *J*(*τ*_*E*_, *τ*_2_) is greater than *J*(*τ*_*E*_, *τ*_1_) ensure that *τ*_1 _cells are more likely to be close to the extracellular medium and to surround the *τ*_2 _cells. It is reflected by the extracellular surface tensions: *γ*_1*E *_= 0 and *γ*_2*E *_= 1. The results are displayed in Figure 4.

At the bottom of Figures 2, 3, 4, the evolution of the energy as well as the rate of acceptance is plotted as a function of the number cycles of Metropolis-Hastings algorithm. These curves exhibite a flat profile, which suggests that stationarity was indeed reached.

### Statistical estimation of the adhesion strength parameter

In this section, we study the sensitivity of simulation results to the adhesion strength parameter *θ*, and we report the performances of the maximum pseudo-likelihood estimator θ^
 MathType@MTEF@5@5@+=feaafiart1ev1aaatCvAUfKttLearuWrP9MDH5MBPbIqV92AaeXatLxBI9gBaebbnrfifHhDYfgasaacH8akY=wiFfYdH8Gipec8Eeeu0xXdbba9frFj0=OqFfea0dXdd9vqai=hGuQ8kuc9pgc9s8qqaq=dirpe0xb9q8qiLsFr0=vr0=vr0dc8meaabaqaciaacaGaaeqabaqabeGadaaakeaaiiGacuWF4oqCgaqcaaaa@2E79@.

To assess the influence of *θ *on simulations, three values were tested: *θ *= 1, *θ *= 5 and *θ *= 10. The results are presented for simulations of checkerboard, cell sorting and engulfment patterns. In each case, the interaction intensities were set as in the previous paragraph.

**Figure 5:**
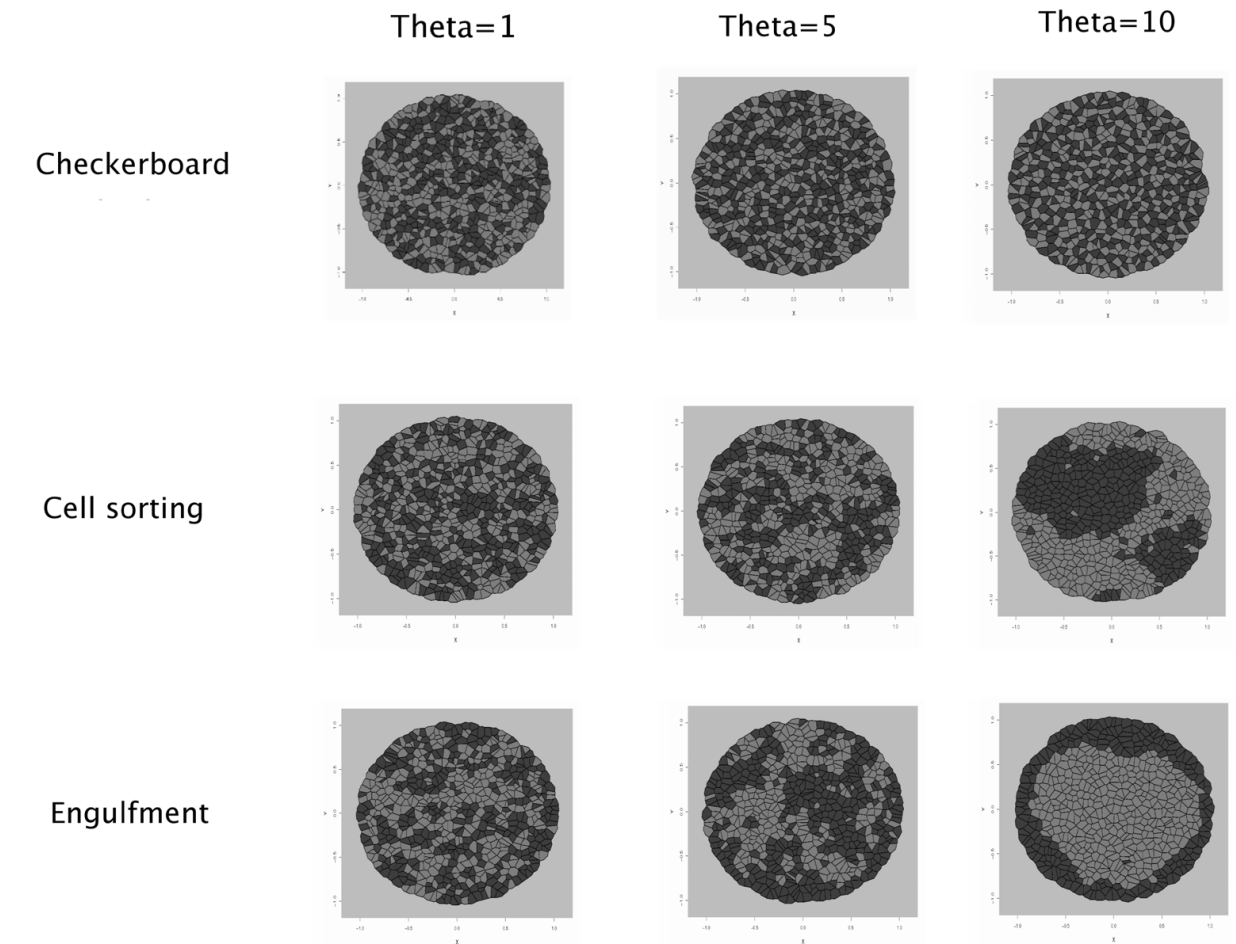
Influence of *θ *in simulations. Final configurations using three different values for *θ*. Simulations gradually corresponds to either a checkerboard, large clusters or engulfment.

We ran the Metropolis algorithm for 100,000 cycles. This number is sufficient to provide a flat profile of energy and rate of acceptance. The final configurations, in checkerboard, cell sorting and engulfment, are displayed in Figure 5. Either for checkerboard or for cell sorting simulations, we observe a gradual evolution when *θ *increases. For *θ *= 1, the marks seem to be randomly distributed, for *θ *= 5 a small inhibition is visible in the checkerboard simulation, small clusters appear in the cell sorting pattern and black cells start to surround white cells in the engulfment simulation. Finally, for *θ *= 10 the stronger inhibition between cells with the same types provides a more pronounced checkerboard pattern, larger clusters are obtained in cell sorting and black cells completely engulf white cells.

For each value of *θ*, 100 replicates of cell sorting, checkerboard and engulfment were generated from which the mean and the variance of θ^
 MathType@MTEF@5@5@+=feaafiart1ev1aaatCvAUfKttLearuWrP9MDH5MBPbIqV92AaeXatLxBI9gBaebbnrfifHhDYfgasaacH8akY=wiFfYdH8Gipec8Eeeu0xXdbba9frFj0=OqFfea0dXdd9vqai=hGuQ8kuc9pgc9s8qqaq=dirpe0xb9q8qiLsFr0=vr0=vr0dc8meaabaqaciaacaGaaeqabaqabeGadaaakeaaiiGacuWF4oqCgaqcaaaa@2E79@ were estimated. Each replicate consisted in 100,000 cycles started from independent initial configurations and sampled from uniform distributions. The number of active cells was sampled from the interval [500,1500]. Cells were uniformly located within the unit disk and types were uniformly assigned to each cell. Table 1 summarizes the results obtained for *θ *in the range [1, 20]. For cell sorting, the bias is weak for all values of *θ*, while for checkerboard the bias seems to be slightly higher. The results are similar regarding the variance. It is higher for checkerboard than for cell sorting. Under the engulfment model, the estimator θ^
 MathType@MTEF@5@5@+=feaafiart1ev1aaatCvAUfKttLearuWrP9MDH5MBPbIqV92AaeXatLxBI9gBaebbnrfifHhDYfgasaacH8akY=wiFfYdH8Gipec8Eeeu0xXdbba9frFj0=OqFfea0dXdd9vqai=hGuQ8kuc9pgc9s8qqaq=dirpe0xb9q8qiLsFr0=vr0=vr0dc8meaabaqaciaacaGaaeqabaqabeGadaaakeaaiiGacuWF4oqCgaqcaaaa@2E79@ seemed to systematically slightly overestimate *θ*. Variance under the engulfment model is of the same order as the variance in cell sorting. Finally, in the three model, the variance increased as *θ *increased. The estimates can be considered as accurate for moderate values of *θ *(≈ 10), as the pseudo-likelihood may provide significant bias in cases of strong interaction [38].

**Table 1:** Mean and Variance of θ^
 MathType@MTEF@5@5@+=feaafiart1ev1aaatCvAUfKttLearuWrP9MDH5MBPbIqV92AaeXatLxBI9gBaebbnrfifHhDYfgasaacH8akY=wiFfYdH8Gipec8Eeeu0xXdbba9frFj0=OqFfea0dXdd9vqai=hGuQ8kuc9pgc9s8qqaq=dirpe0xb9q8qiLsFr0=vr0=vr0dc8meaabaqaciaacaGaaeqabaqabeGadaaakeaaiiGacuWF4oqCgaqcaaaa@2E79@, maximum of Pseudo-likelihood, for checkerboard, cell sorting and engulf-mentsimulations. The evaluation was achieved using 100 simulations of each case.

	Checkerboard		Cell Sorting		Engulfment	
	Mean	Variance	Mean	Variance	Mean	Variance

*θ *= 1	0.99	0.39	1.12	0.34	1.27	0.32
*θ *= 3	3.34	0.48	3.03	0.43	3.08	0.41
*θ *= 5	5.15	0.92	5.37	0.45	5.23	0.48
*θ *= 8	8.42	1.03	8.11	0.92	8.21	0.67
*θ *= 10	10.58	1.36	10.26	0.67	10.22	1.02
*θ *= 12	12.12	1.55	12.14	0.92	12.18	0.94
*θ *= 15	15.51	1.72	14.94	1.64	15.48	1.12
*θ *= 20	19.52	2.15	20.08	1.51	20.28	1.82

### Experimental data

**Figure 6:**
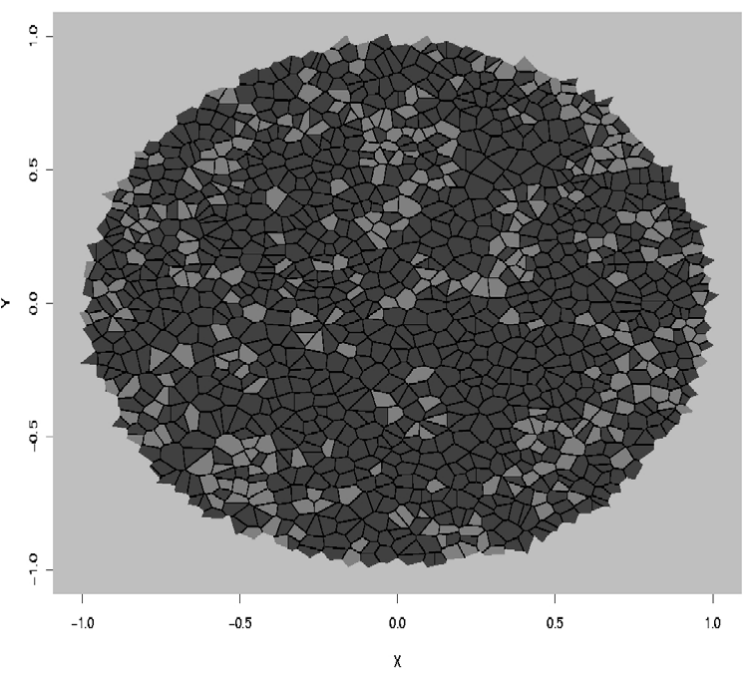
Experimental data. The statistical procedure was conducted using the interactions parameters for cell sorting patterns: *J*(*τ*_1_, *τ*_1_) = 1, *J*(*τ*_2_, *τ*_2_) = 1, *J*(*τ*_1_, *τ*_2_) = 0, *J*(*τ*_1_, *τ*_*E*_) = 0, *J*(*τ*_2_, *τ*_*E*_) = 0 and *J*(*τ*_*E*_, *τ*_*E*_) = 0. *λ *was set to 10,000. We obtained θ^
 MathType@MTEF@5@5@+=feaafiart1ev1aaatCvAUfKttLearuWrP9MDH5MBPbIqV92AaeXatLxBI9gBaebbnrfifHhDYfgasaacH8akY=wiFfYdH8Gipec8Eeeu0xXdbba9frFj0=OqFfea0dXdd9vqai=hGuQ8kuc9pgc9s8qqaq=dirpe0xb9q8qiLsFr0=vr0=vr0dc8meaabaqaciaacaGaaeqabaqabeGadaaakeaaiiGacuWF4oqCgaqcaaaa@2E79@ ≈ 5,27.

Estimation of the adhesion strength was also performed on a real data example. We used data from Pizem *et al*. ([39]), who measured survivin and beta-catenin markers in Human medulloblastoma. These markers are known to be involved in complexes that regulate adhesion between contiguous cells. An image analysis, analogous to the analysis performed in [40], was achieved to extract the locations of cell nuclei and the levels of expression of markers in cells. The expression levels were used to define cell types as displayed in Figure 6. The resulting image is relevant to a cell sorting pattern, and we used the set of *J *parameters that corresponded to this pattern.

**Figure 7:**
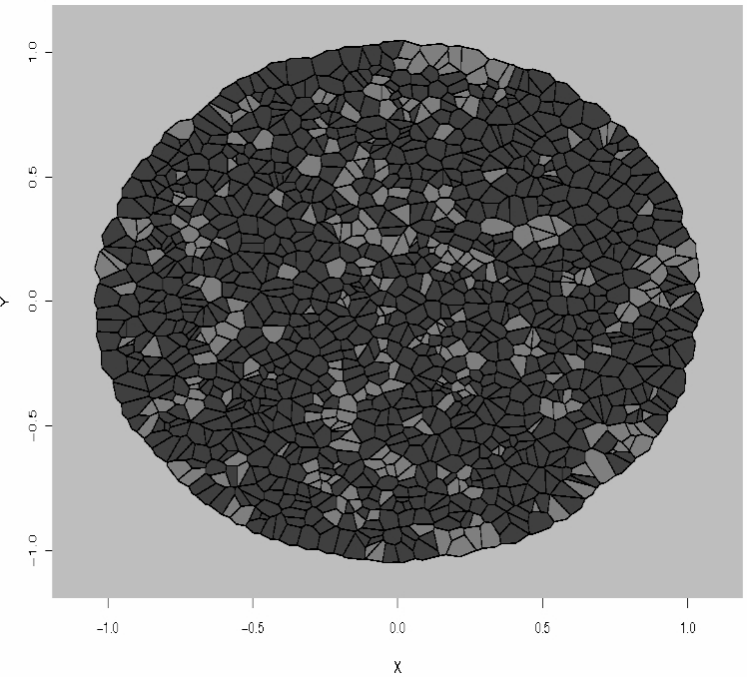
Simulated data. Using the interactions parameters for cell sorting patterns: *J*(*τ*_1_, *τ*_1_) = 1, *J*(*τ*_2_, *τ*_2_) = 1, *J*(*τ*_1_, *τ*_2_) = 0, *J*(*τ*_1_, *τ*_*E*_) = 0, *J*(*τ*_2_, *τ*_*E*_) = 0 and *J*(*τ*_*E*_, *τ*_*E*_) = 0. The parameter *θ *used for the simulation is the estimated value from experimental data *θ *= 5.27 (see Figure 6). The estimated interaction parameter θ^
 MathType@MTEF@5@5@+=feaafiart1ev1aaatCvAUfKttLearuWrP9MDH5MBPbIqV92AaeXatLxBI9gBaebbnrfifHhDYfgasaacH8akY=wiFfYdH8Gipec8Eeeu0xXdbba9frFj0=OqFfea0dXdd9vqai=hGuQ8kuc9pgc9s8qqaq=dirpe0xb9q8qiLsFr0=vr0=vr0dc8meaabaqaciaacaGaaeqabaqabeGadaaakeaaiiGacuWF4oqCgaqcaaaa@2E79@ ≈ 5.51.

The estimate of *θ *was computed as θ^
 MathType@MTEF@5@5@+=feaafiart1ev1aaatCvAUfKttLearuWrP9MDH5MBPbIqV92AaeXatLxBI9gBaebbnrfifHhDYfgasaacH8akY=wiFfYdH8Gipec8Eeeu0xXdbba9frFj0=OqFfea0dXdd9vqai=hGuQ8kuc9pgc9s8qqaq=dirpe0xb9q8qiLsFr0=vr0=vr0dc8meaabaqaciaacaGaaeqabaqabeGadaaakeaaiiGacuWF4oqCgaqcaaaa@2E79@ ≈ 5.27. This value provides evidence that the model is able to detect large clusters (black cell clusters here) and that white cells may be surrounded by black cells. The estimated value θ^
 MathType@MTEF@5@5@+=feaafiart1ev1aaatCvAUfKttLearuWrP9MDH5MBPbIqV92AaeXatLxBI9gBaebbnrfifHhDYfgasaacH8akY=wiFfYdH8Gipec8Eeeu0xXdbba9frFj0=OqFfea0dXdd9vqai=hGuQ8kuc9pgc9s8qqaq=dirpe0xb9q8qiLsFr0=vr0=vr0dc8meaabaqaciaacaGaaeqabaqabeGadaaakeaaiiGacuWF4oqCgaqcaaaa@2E79@ was then tested as input to the simulation algorithm, and the resulting spatial pattern is displayed in Figure 7. Comparing the real tissue and the cell sorting pattern simulated with the estimated interaction strength makes clear that the model provides a good fit to the data and that *θ *estimation is consistent.

## Conclusion

In this study, we presented an approach to cell sorting based on marked point processes theory. It proposes a continuous geometry for tissues using a Dirichlet tessellation and an energy functional expressed as the sum of two terms: an interaction term between two contiguous cells weighted by the length of the membrane and a cell shape constraint term. Such models, where interactions are weighted by the length of the membrane, have already been considered in the literature, first by Sulsky *et al*. [20] and next by Graner and Sawada [21]. Based on Honda's studies that showed that the geometry of Dirichlet cells was in agreement with biological tissues [41,42], these earlier models also used a continuous geometry of cells. These authors were interested in formulating a dynamical model which determines not only the equilibrium state but the path from the initial state to final state. These two approaches introduced systems of differential equations to simulate cell patterns.

Although the previous approaches contained the main ingredients to model simulation, they were not well-adapted to perform statistical estimation of interaction parameters. Furthermore, Graner and Sawada reported two limitations of their approach. First, because the GS model is not stochastic, it does not explore the set of possible configurations ([21], p.497, l.10). Next Graner and Sawada stressed that their simulation algorithm suffers from instability because of its lack of theoretical control ([21], p.497, l.15). Graner and Glazier proposed Boltzmann dynamics and were interested in the time needed to achieve desired configurations. However, there is no warranty that their Markov chain has correct mixing properties, and the sensitivity of their method to the discretization scale remains to be studied. Because of discretization, detailed balance condition and cell connexity did not seem to hold in the GG model. GG's approach cannot be easily adapted to define inference procedures.

Our study is not the first attempt to propose statistical procedures for estimating interaction strength parameters in tissues. In [13], two statistics have been introduced to measure the degree of spatial cell sorting in a tissue where cells are of types black and white. Cell sorting can be quantified by the fraction of black cells in the nearest neighborhood of single black cell and the number of isolated black cells. Although these two statistics have been recently used to study the role of cadherins in tissue segregation [43], their practical application requires cells to be pixels within a lattice ([13] and [43]). Their capacity to quantify cell sorting has been studied using a cell-lattice model where all cells have the same geometry, hypothesis which does not fit with the zipper-like structure of cadherins [25].

In contrast to these approaches, the mathematical background of marked point processes allows the establishment of a statistical framework. In this study, we have shown that our model was able to reproduce biologically relevant cell patterns such as checkerboard, cell sorting and engulfment. Checkerboard pattern formation was investigated in a simulation study of the sexual maturation of the avian oviduct epithelium [44]. Cell sorting is a standard pattern of mixed heterotypic aggregates. Experimental observations of this phenomena were reported by Takeuchi *et al*. [45] and Armstrong [1]. Engulfment of a tissue by another one was studied by Armstrong [1] and Foty *et al*. [46]. This phenomenon is a direct consequence of adhesion processes between the two cell types and the extracellular medium. These cell patterns were also simulated by pioneering studies ([17,20,21]).

Furthermore, the present model has been built so that it includes the strength of cell-cell adhesion as a statistical parameter. We proposed and validated an inference procedure based on the pseudo-likelihood. The statistical errors remain small in cell sorting simulations. In checkerboard simulations, bias and variance are slightly higher than for cell sorting but still reasonable. The bias is also weak in engulfment simulations. Further improvements of this approach would require a longer study of the properties of the point process model. In particular, the other interaction parameters can also be estimated in the same way that *θ *is. Although we did not assess the performances of these estimators, we believe that they would be useful for analyzing tissues arrays, as generated by high-throughput cancer studies [47].

## Competing interests

The author(s) declare that they have no competing interests.

## Authors' contributions

OF and ME both provided the basic ideas of the project. ME was responsible for the development of the proposed method and carried out the simulation analysis. ME and OF equally contributed to the writing of the manuscript. All authors read and approved the final manuscript.

## Appendix

### Appendix 1

In this section we prove that the point process introduced in this paper is a *nearest-neighbour markov point process *which has been defined in [32] (p.107 Definition 4.9).

We use Theorem 4.13 (p.108) in [32] (analogue of the Hammersley-Clifford-Ripley-Kelly theorem [48] for nearest-neighbour markov point processes) which is as follows

**Theorem 1 ***Let H be an hereditary subset of the set of finite configurations in *X
 MathType@MTEF@5@5@+=feaafiart1ev1aaatCvAUfKttLearuWrP9MDH5MBPbIqV92AaeXatLxBI9gBaebbnrfifHhDYfgasaacH8akY=wiFfYdH8Gipec8Eeeu0xXdbba9frFj0=OqFfea0dXdd9vqai=hGuQ8kuc9pgc9s8qqaq=dirpe0xb9q8qiLsFr0=vr0=vr0dc8meaabaqaciaacaGaaeqabaqabeGadaaakeGabaaBymrtHrhAL1wy0L2yHvtyaeHbnfgDOvwBHrxAJfwnaGabaiab=Dr8ybaa@38D5@*× M. Let ~_*ϕ *_be a neighbour relation with consistency conditions (C1)–(C2) (4.7 p.106 in *[32]*) hold, ϕ ∈ H. Then a function g *: *H *→ [0, ∞) *is a Markov function if and only if*

g(x)=∏φ⊂ϕΨ(φ|ϕ)
 MathType@MTEF@5@5@+=feaafiart1ev1aaatCvAUfKttLearuWrP9MDH5MBPbIqV92AaeXatLxBI9gBaebbnrfifHhDYfgasaacH8akY=wiFfYdH8Gipec8Eeeu0xXdbba9frFj0=OqFfea0dXdd9vqai=hGuQ8kuc9pgc9s8qqaq=dirpe0xb9q8qiLsFr0=vr0=vr0dc8meaabaqaciaacaGaaeqabaqabeGadaaakeaacqWGNbWzcqGGOaakcqWG4baEcqGGPaqkcqGH9aqpdaqeqbqaaiabfI6azjabcIcaOGGaciab=z8aMjabcYha8jab=v9aQjabcMcaPaWcbaGae8NXdyMaeyOGIWSae8x1dOgabeqdcqGHpis1aaaa@4205@

*for all ϕ *∈ *H, where Ψ is an interaction function*.

As proved in [32] (Appendix A1, p116), the set of Dirichlet configurations is hereditary and satisfies properties (C1) and (C2) (see paragraphs 2.5 p94 and 4.7 p106 in [32]).

Let *ϕ *be a finite configuration in X
 MathType@MTEF@5@5@+=feaafiart1ev1aaatCvAUfKttLearuWrP9MDH5MBPbIqV92AaeXatLxBI9gBaebbnrfifHhDYfgasaacH8akY=wiFfYdH8Gipec8Eeeu0xXdbba9frFj0=OqFfea0dXdd9vqai=hGuQ8kuc9pgc9s8qqaq=dirpe0xb9q8qiLsFr0=vr0=vr0dc8meaabaqaciaacaGaaeqabaqabeGadaaakeGabaaBymrtHrhAL1wy0L2yHvtyaeHbnfgDOvwBHrxAJfwnaGabaiab=Dr8ybaa@38D5@ and ~_*ϕ *_its Dirichlet neighbourhood. Let Ψ be a function defined over all cliques *φ *∈ *ϕ *as follows

Ψ({xi,τi})=exp(−λh(Dir(xi),τi))Z(θ)>0 for each point (xi,τi)∈ϕ
 MathType@MTEF@5@5@+=feaafiart1ev1aaatCvAUfKttLearuWrP9MDH5MBPbIqV92AaeXatLxBI9gBaebbnrfifHhDYfgasaacH8akY=wiFfYdH8Gipec8Eeeu0xXdbba9frFj0=OqFfea0dXdd9vqai=hGuQ8kuc9pgc9s8qqaq=dirpe0xb9q8qiLsFr0=vr0=vr0dc8meaabaqaciaacaGaaeqabaqabeGadaaakeaacqqHOoqwcqGGOaakcqGG7bWEcqWG4baEdaWgaaWcbaGaemyAaKgabeaakiabcYcaSGGaciab=r8a0naaBaaaleaacqWGPbqAaeqaaOGaeiyFa0NaeiykaKIaeyypa0ZaaSaaaeaacqWGLbqzcqWG4baEcqWGWbaCcqGGOaakcqGHsislcqWF7oaBcqWGObaAcqGGOaakcqqGebarcqqGPbqAcqqGYbGCcqGGOaakcqWG4baEdaWgaaWcbaGaemyAaKgabeaakiabcMcaPiabcYcaSiab=r8a0naaBaaaleaacqWGPbqAaeqaaOGaeiykaKIaeiykaKcabaGaemOwaOLaeiikaGIae8hUdeNaeiykaKcaaiabg6da+iabicdaWiabbccaGiabbAgaMjabb+gaVjabbkhaYjabbccaGiabbwgaLjabbggaHjabbogaJjabbIgaOjabbccaGiabbchaWjabb+gaVjabbMgaPjabb6gaUjabbsha0jabbccaGiabcIcaOiabdIha4naaBaaaleaacqWGPbqAaeqaaOGaeiilaWIae8hXdq3aaSbaaSqaaiabdMgaPbqabaGccqGGPaqkcqGHiiIZcqWFvpGAaaa@79DA@

Ψ(i,j)=exp(−θ|Dir(xi∩xj)|J(τi,τj))Z(θ)>0 for each couple {(xi,τi),(xj,τj)} such as xi~ϕxj
 MathType@MTEF@5@5@+=feaafiart1ev1aaatCvAUfKttLearuWrP9MDH5MBPbIqV92AaeXatLxBI9gBaebbnrfifHhDYfgasaacH8akY=wiFfYdH8Gipec8Eeeu0xXdbba9frFj0=OqFfea0dXdd9vqai=hGuQ8kuc9pgc9s8qqaq=dirpe0xb9q8qiLsFr0=vr0=vr0dc8meaabaqaciaacaGaaeqabaqabeGadaaakeaacqqHOoqwcqGGOaakcqWGPbqAcqGGSaalcqWGQbGAcqGGPaqkcqGH9aqpdaWcaaqaaiabdwgaLjabdIha4jabdchaWjabcIcaOiabgkHiTGGaciab=H7aXjabcYha8jabbseaejabbMgaPjabbkhaYjabcIcaOiabdIha4naaBaaaleaacqWGPbqAaeqaaOGaeyykICSaemiEaG3aaSbaaSqaaiabdQgaQbqabaGccqGGPaqkcqGG8baFcqWGkbGscqGGOaakcqWFepaDdaWgaaWcbaGaemyAaKgabeaakiabcYcaSiab=r8a0naaBaaaleaacqWGQbGAaeqaaOGaeiykaKIaeiykaKcabaGaemOwaOLaeiikaGIae8hUdeNaeiykaKcaaiabg6da+iabicdaWiabbccaGiabbAgaMjabb+gaVjabbkhaYjabbccaGiabbwgaLjabbggaHjabbogaJjabbIgaOjabbccaGiabbogaJjabb+gaVjabbwha1jabbchaWjabbYgaSjabbwgaLjabbccaGiabcUha7jabcIcaOiabdIha4naaBaaaleaacqWGPbqAaeqaaOGaeiilaWIae8hXdq3aaSbaaSqaaiabdMgaPbqabaGccqGGPaqkcqGGSaalcqGGOaakcqWG4baEdaWgaaWcbaGaemOAaOgabeaakiabcYcaSiab=r8a0naaBaaaleaacqWGQbGAaeqaaOGaeiykaKIaeiyFa0NaeeiiaaIaee4CamNaeeyDauNaee4yamMaeeiAaGMaeeiiaaIaeeyyaeMaee4CamNaeeiiaaIaemiEaG3aaSbaaSqaaiabdMgaPbqabaGccqGG+bGFdaWgaaWcbaGae8x1dOgabeaakiabdIha4naaBaaaleaacqWGQbGAaeqaaaaa@9CDD@

Ψ (clique) = 1 for all cliques with three or more points

As mentioned in the section "A new model for DAH", one can remark that for each marked cell (*x*_*i*_, *τ*_*i*_) in a configuration *ϕ *∈ X
 MathType@MTEF@5@5@+=feaafiart1ev1aaatCvAUfKttLearuWrP9MDH5MBPbIqV92AaeXatLxBI9gBaebbnrfifHhDYfgasaacH8akY=wiFfYdH8Gipec8Eeeu0xXdbba9frFj0=OqFfea0dXdd9vqai=hGuQ8kuc9pgc9s8qqaq=dirpe0xb9q8qiLsFr0=vr0=vr0dc8meaabaqaciaacaGaaeqabaqabeGadaaakeGabaaBymrtHrhAL1wy0L2yHvtyaeHbnfgDOvwBHrxAJfwnaGabaiab=Dr8ybaa@38D5@ × ℳ
 MathType@MTEF@5@5@+=feaafiart1ev1aaatCvAUfKttLearuWrP9MDH5MBPbIqV92AaeXatLxBI9gBaebbnrfifHhDYfgasaacH8akY=wiFfYdH8Gipec8Eeeu0xXdbba9frFj0=OqFfea0dXdd9vqai=hGuQ8kuc9pgc9s8qqaq=dirpe0xb9q8qiLsFr0=vr0=vr0dc8meaabaqaciaacaGaaeqabaqabeGadaaakeGabaaBymrtHrhAL1wy0L2yHvtyaeHbnfgDOvwBHrxAJfwnaGabaiab=ntinbaa@3816@

Ψ({xi,τi})=exp(−λh(Dir(xi),τi))Z(θ)>0
 MathType@MTEF@5@5@+=feaafiart1ev1aaatCvAUfKttLearuWrP9MDH5MBPbIqV92AaeXatLxBI9gBaebbnrfifHhDYfgasaacH8akY=wiFfYdH8Gipec8Eeeu0xXdbba9frFj0=OqFfea0dXdd9vqai=hGuQ8kuc9pgc9s8qqaq=dirpe0xb9q8qiLsFr0=vr0=vr0dc8meaabaqaciaacaGaaeqabaqabeGadaaakeaacqqHOoqwcqGGOaakcqGG7bWEcqWG4baEdaWgaaWcbaGaemyAaKgabeaakiabcYcaSGGaciab=r8a0naaBaaaleaacqWGPbqAaeqaaOGaeiyFa0NaeiykaKIaeyypa0ZaaSaaaeaacqWGLbqzcqWG4baEcqWGWbaCcqGGOaakcqGHsislcqWF7oaBcqWGObaAcqGGOaakcqqGebarcqqGPbqAcqqGYbGCcqGGOaakcqWG4baEdaWgaaWcbaGaemyAaKgabeaakiabcMcaPiabcYcaSiab=r8a0naaBaaaleaacqWGPbqAaeqaaOGaeiykaKIaeiykaKcabaGaemOwaOLaeiikaGIae8hUdeNaeiykaKcaaiabg6da+iabicdaWaaa@5A2E@

, and for each couple {(*x*_*i*_, *τ*_*i*_), (*x*_*j*_, *τ*_*j*_)}, such as *x*_*i *_~_*ϕ *_*x*_*j*_

Ψ(i,j)=exp(−θ|Dir(xi∩xj)|J(τi,τj))Z(θ)>0
 MathType@MTEF@5@5@+=feaafiart1ev1aaatCvAUfKttLearuWrP9MDH5MBPbIqV92AaeXatLxBI9gBaebbnrfifHhDYfgasaacH8akY=wiFfYdH8Gipec8Eeeu0xXdbba9frFj0=OqFfea0dXdd9vqai=hGuQ8kuc9pgc9s8qqaq=dirpe0xb9q8qiLsFr0=vr0=vr0dc8meaabaqaciaacaGaaeqabaqabeGadaaakeaacqqHOoqwcqGGOaakcqWGPbqAcqGGSaalcqWGQbGAcqGGPaqkcqGH9aqpdaWcaaqaaiabdwgaLjabdIha4jabdchaWjabcIcaOiabgkHiTGGaciab=H7aXjabcYha8jabbseaejabbMgaPjabbkhaYjabcIcaOiabdIha4naaBaaaleaacqWGPbqAaeqaaOGaeyykICSaemiEaG3aaSbaaSqaaiabdQgaQbqabaGccqGGPaqkcqGG8baFcqWGkbGscqGGOaakcqWFepaDdaWgaaWcbaGaemyAaKgabeaakiabcYcaSiab=r8a0naaBaaaleaacqWGQbGAaeqaaOGaeiykaKIaeiykaKcabaGaemOwaOLaeiikaGIae8hUdeNaeiykaKcaaiabg6da+iabicdaWaaa@5E4E@

This shows that Ψ is an *interaction function *in the sense of [32] (definition 4.11 p108). One can easily note that ∏_*c*∈*cliques*(*ϕ*) _Ψ(*c*) = *f *(*ϕ*, *θ*). Then Theorem 1 holds for the interaction function proposed in this paper which leads to *f *is a Markov function.

Since the neighbourhood depends on the configuration, our point process is a *nearest-neighbour markov point process *as defined in [32] (Definition 4.9 p107).

### Appendix 2

Here is the description of one iteration in the MCMC algorithm proposed in this paper. Let us denote *ϕ *the configuration just before the current iteration and *ψ *the proposed new configuration.

• With probability *p*(*ϕ*) – Displacement

- One point {*x*_*i*_, *τ*_*i*_} is chosen with probability *d*(*ϕ*, {*x*_*i*_, *τ*_*i*_})

- The proposal distribution for the moving point is *b*(*ϕ*, {*x*, *τ*})

- *ψ *= *ϕ *⊂ {*x*_*i*_, *τ*_*i*_} ∪ {*x*, *τ*}

• Else with probability (1 - *p*(*ϕ*))*q*(*ϕ*) – Insertion

- The proposal distribution for the new point is *b*(*ϕ*, {*x*, *τ*})

- *ψ *= *ϕ *∪ {*x*, *τ*}

• Else with probability (1 - *p*(*ϕ*))(1 - *q*(*ϕ*)) – Deletion

- The deleted point {*x*_*i*_, *τ*_*i*_} is chosen with probability *d*(*ϕ*, {*x*_*i*_, *τ*_*i*_})

- *ψ *= *ϕ *⊂ {*x*_*i*_, *τ*_*i*_}

• The proposal configuration *ψ *is accepted with the acceptance probability A [31], as follows

*A*(*ψ *|*ϕ*) = min(1, *f*(*ψ*)/*f*(*ϕ*))

In the algorithm proposed in this paper, we used

*p*(*ϕ*) = 1/2 and *q*(*ϕ*) = 1/2 for all *ϕ *X
 MathType@MTEF@5@5@+=feaafiart1ev1aaatCvAUfKttLearuWrP9MDH5MBPbIqV92AaeXatLxBI9gBaebbnrfifHhDYfgasaacH8akY=wiFfYdH8Gipec8Eeeu0xXdbba9frFj0=OqFfea0dXdd9vqai=hGuQ8kuc9pgc9s8qqaq=dirpe0xb9q8qiLsFr0=vr0=vr0dc8meaabaqaciaacaGaaeqabaqabeGadaaakeGabaaBymrtHrhAL1wy0L2yHvtyaeHbnfgDOvwBHrxAJfwnaGabaiab=Dr8ybaa@38D5@ × *M*

*d*(*ϕ*, {*x*_*i*_, *τ*_*i*_}) = 1/*n *for all {*x*_*i*_, *τ*_*i*_} ∈ *ϕ *and where *n *is the number of points in *ϕ *b(ϕ,{x,τ})=1ρ(X)ϱ(M)
 MathType@MTEF@5@5@+=feaafiart1ev1aaatCvAUfKttLearuWrP9MDH5MBPbIqV92AaeXatLxBI9gBaebbnrfifHhDYfgasaacH8akY=wiFfYdH8Gipec8Eeeu0xXdbba9frFj0=OqFfea0dXdd9vqai=hGuQ8kuc9pgc9s8qqaq=dirpe0xb9q8qiLsFr0=vr0=vr0dc8meaabaqaciaacaGaaeqabaqabeGadaaakeaacqWGIbGycqGGOaakiiGacqWFvpGAcqGGSaalcqGG7bWEcqWG4baEcqGGSaalcqWFepaDcqGG9bqFcqGGPaqkcqGH9aqpdaWcaaqaaiabigdaXaqaceaaSHHae8xWdiNaeiikaGYenfgDOvwBHrxAJfwnHbqeg0uy0HwzTfgDPnwy1aaceaGae43fXJLaeiykaKIaeSy==7gcbaGae0hkaGccbiGaeWxta0Kae0xkaKcaaaaa@50E5@ where *ρ *× ϱ is the intensity of the underlying marked Poisson process
